# Catching a Walker in the Act—DNA Partitioning by ParA Family of Proteins

**DOI:** 10.3389/fmicb.2022.856547

**Published:** 2022-05-26

**Authors:** Dipika Mishra, Ramanujam Srinivasan

**Affiliations:** ^1^School of Biological Sciences, National Institute of Science Education and Research, Bhubaneswar, India; ^2^Homi Bhabha National Institutes, Mumbai, India

**Keywords:** DNA segregation, plasmid, ParA, Walker A motif, ParB

## Abstract

Partitioning the replicated genetic material is a crucial process in the cell cycle program of any life form. In bacteria, many plasmids utilize cytoskeletal proteins that include ParM and TubZ, the ancestors of the eukaryotic actin and tubulin, respectively, to segregate the plasmids into the daughter cells. Another distinct class of cytoskeletal proteins, known as the Walker A type Cytoskeletal ATPases (WACA), is unique to Bacteria and Archaea. ParA, a WACA family protein, is involved in DNA partitioning and is more widespread. A centromere-like sequence *parS*, in the DNA is bound by ParB, an adaptor protein with CTPase activity to form the segregation complex. The ParA ATPase, interacts with the segregation complex and partitions the DNA into the daughter cells. Furthermore, the Walker A motif-containing ParA superfamily of proteins is associated with a diverse set of functions ranging from DNA segregation to cell division, cell polarity, chemotaxis cluster assembly, cellulose biosynthesis and carboxysome maintenance. Unifying principles underlying the varied range of cellular roles in which the ParA superfamily of proteins function are outlined. Here, we provide an overview of the recent findings on the structure and function of the ParB adaptor protein and review the current models and mechanisms by which the ParA family of proteins function in the partitioning of the replicated DNA into the newly born daughter cells.

## Introduction

The genetic material in all organisms needs to be equipartitioned during each round of cell division. This mechanism has been very well-studied in eukaryotes. The initial study on eukaryotic chromosome segregation dates back to the later part of the nineteenth century with the discovery of thread-like structures within the nucleus of the stained newt cells. These thread-like structures, observed with a light microscope, were named chromatin (Flemming, 1882). Subsequently, the entire mechanism of eukaryotic chromosome segregation was characterized. It is now known to be carried out by spindle fibers, composed of microtubules that pull apart the chromosomes and assist in segregation (Scholey et al., 2003; Kline-Smith and Walczak, 2004). This mechanism is very well-coordinated and is programmed into four different phases of the cell cycle- S, G1, M, and G2 (Cooper, 2000; Walczak et al., 2010).

Unlike the eukaryotic genetic material, the prokaryotic DNA is not encased within a nuclear membrane. Instead, it spreads over the entire cytosol of bacteria and is called the nucleoid. The term “nucleoid” was first coined by Piekarski (1937). With the progression of the cell cycle, the nucleoid changes its shape to a bilobed one and soon segregates into two daughter cells (Zimmerman, 2003; Yamaichi and Niki, 2004; Fisher et al., 2013). The genetic material in the eukaryotes is held together by histone and cohesion proteins (Losada and Hirano, 2005; Nasmyth and Haering, 2005). However, in the case of prokaryotes, the chromosomes are held together by DNA binding proteins called Nucleoid Associated Proteins (NAP) (Kar et al., 2005; Badrinarayanan et al., 2015) that help in chromosomal compaction and organization of domains known as the high-density regions (HDRs). These NAPs include HU, HNS, Fis, and IHF (Ali Azam et al., 1999; Verma et al., 2019). The nucleoid occupies a major proportion of the bacterial cytosol and plays an integral and decisive role in positioning the cytokinetic Z-ring (Harry et al., 1999; Yu and Margolin, 1999; Harry, 2001; Sun and Margolin, 2001; Bernhardt and de Boer, 2005; Rothfield et al., 2005) as well as driving the ParA mediated DNA partitioning (Castaing et al., 2008; Le Gall et al., 2016; McLeod et al., 2017).

DNA segregation in bacteria has been extensively studied using plasmids as a model system. Plasmids are extrachromosomal self-replicating species of DNA that usually encode genes for antibiotic resistance, production of bacteriocins, resistance to heavy metals, ultraviolet light, pathogen virulence factors and many other metabolic functions (Birge, 2006). Plasmids generally vary in size from a few kilobases to hundreds of kilobases, and their geometry is commonly circular or linear. Plasmids have been traditionally classified into different types based on their replication and copy numbers (Million-Weaver and Camps, 2014).

High copy number plasmids are generally small and replicate randomly during the cell cycle. These plasmids can reach up to a 100 copies per cell, and thus random assortment and segregation during cytokinesis can ensure sufficient distribution of these plasmids into two daughter cells (Birge, 2006; Million-Weaver and Camps, 2014). On the contrary, low copy number plasmids with <15 copies per cell cannot solely rely on random distribution for maintenance. Instead, they depend upon dedicated partitioning proteins to distribute them into daughter cells. Further, this is also true for single-copy number plasmids and includes many bacterial genomes. In contrast to the universal nature of the eukaryotic DNA segregation, microbes employ diverse mechanisms to partition their DNA. Active DNA partitioning systems have been broadly classified into Type-I, Type-II, and Type-III according to the protein family of the NTPase in the partitioning system (reviewed in Hayes and Barillà, 2006; Gerdes et al., 2010; Lutkenhaus, 2012). The DNA partitioning functions usually rely upon a member of the actin-like ATPase, tubulin-like GTPase, or Walker A Cytoskeletal ATPase (WACA) family of proteins. The WACA family of proteins is part of a larger group of P-loop ATPases with a deviant Walker A motif and is often referred to as the ParA superfamily (Walker et al., 1982; Motallebi-Veshareh et al., 1990; Koonin, 1993). The ParA superfamily of proteins is also involved in a wide range of diverse cellular functions in bacteria, including DNA segregation. Type-I systems utilize the ParA superfamily of proteins for DNA partitioning. They are found in plasmids such as the F plasmid (Fertility Plasmid that encodes the Fertility factor or the F factor) and in the chromosomes of many bacteria (Abeles et al., 1985; Davis et al., 1992, 1996; Koonin, 1993; Lutkenhaus, 2012). The *Salmonella paratyphi* R1 plasmid carries a Type-II system and contains an actin-like ATPase, ParM, that undergoes insertional polymerisation to push apart the plasmids to the two opposite ends of the cell (Gerdes et al., 2000; Møller-Jensen et al., 2003). The Type-III system is exemplified by the pBToxis plasmid, wherein the tubulin homolog TubZ segregates the two plasmids to the poles (Larsen et al., 2007). The Type-I system is the most widespread, with chromosomes in organisms ranging from archaea to bacterial pathogens and low copy number plasmids carrying virulence factors or multi-antibiotic resistance determinants relying upon the ParA family of proteins for partitioning functions (Motallebi-Veshareh et al., 1990; Koonin, 1993; Lutkenhaus, 2012; Stephens et al., 2020). The low copy number plasmids have been studied for a long time, and together with the recent findings in many bacterial species on chromosomal DNA partitioning, they provide us insights into the mechanism of DNA segregation in prokaryotes.

In this review, we provide a historical overview of the evolution of our understanding of DNA segregation in bacteria, with particular emphasis on the ParA protein from the F plasmid of *E. coli* (ParA_F_) and highlight some of the recent findings. We briefly discuss the various types of plasmid segregation systems (Type-I–Type-III), the functions of ParA and the mechanism by which ParA orchestrates DNA partitioning. The recent findings on the CTPase activity of the centromere binding protein, ParB and its bearings on DNA segregation functions are discussed. We list a few examples of the ParA family of proteins involved in bacterial and archaeal chromosome segregation. Finally, we touch upon the functions of the ParA superfamily of proteins in other diverse cellular processes in bacteria.

## Types of Plasmid Segregation Systems

### Type-I—Walker A-Type ATPases in DNA Partitioning

Most bacterial chromosomes and many low-copy number plasmids depend on the Type-I system for the segregation of DNA. The Type-I system is characterized by the presence of a P-loop ATPase superfamily of protein, known as the ParA/MinD family of ATPases, that have a deviant Walker A box motif KGGXXK[S/T] (Koonin, 1993; Lutkenhaus, 2012). The genetic loci important for partitioning also carry a centromeric repeat sequence, *parS*, and encode a second protein, the centromere binding protein (CBP) or adaptor protein ParB that binds *parS* sites and interacts with ParA as well. The Walker A motif in ParA is directly involved in interactions with the bound ATP molecule. Walker A motifs in the Type-I ParA family differ from the classical Walker A motif in having this additional signature lysine residue (Table 1), and thus the term deviant Walker A motif follows (Motallebi-Veshareh et al., 1990; Hayes, 2000; Lutkenhaus and Sundaramoorthy, 2003; Wendler et al., 2012). A second motif, the B box, characterized by conserved negatively charged residue (D/E), plays an important role in Mg^2+^ coordination and ATP hydrolysis (Fung et al., 2001; Schumacher, 2008). Mutations in the conserved Walker A box lead to the loss of plasmids and point to a key role for ATP hydrolysis in mediating segregation (Ebersbach and Gerdes, 2001; Fung et al., 2001; Libante et al., 2001; Barillà et al., 2005; Pratto et al., 2008). The Type-I system is further sub-divided into Type-Ia and Type-Ib based on the structure of ParA (Hayes, 2000; Schumacher, 2008).

**Table 1:** The deviant Walker A motif in different members of P loop ATPase.

**Protein**	**Deviant Walker A motif** (KGGXXK**[S/T])**
ParA_F_ (SopA)	KGGVYKT
ParA_Bsu_ & ParA_Hpy_ [Soj (*B. subtilis* & *H. pylori*)]	KGGVGKT
MinD	KGGVGKT
FleN	KGGVGKT
PpfA	KGGVGKT
FlhG	KGGVGKS
MipZ	KGGAGKS
ParI	KGGVGKS
McdA	SGGQGKT
BcsQ	RGGVGTT

*The signature lysine is marked in red and the catalytic lysine is marked in blue*.

**Figure 1:**
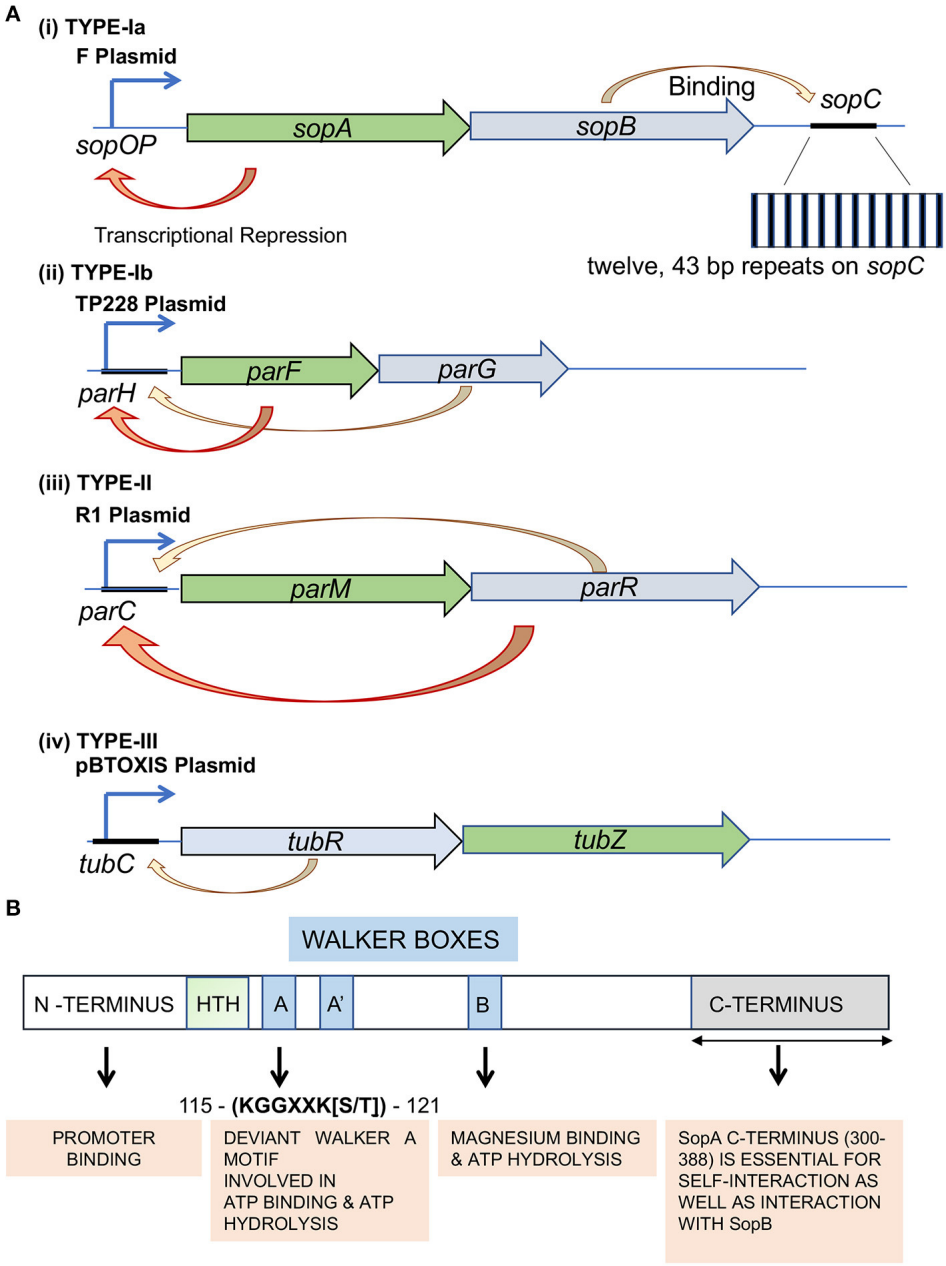
Types of plasmid partitioning systems and domain organization in the ParA family of proteins Type-I system. **(A)** Genetic organization of Type-Ia, Type-Ib, Type-II, and Type-III partition loci. Genes encoding the ATPase (green) and adaptor protein (blue) are indicated. The auto-repression activity is represented by an orange arrow. F plasmid, TP228 plasmid, R plasmid and pBToxis plasmid are used as representatives of (i) Type-Ia, (ii) Type-Ib, (iii) Type-II and (iv) Type-III segregation mechanisms, respectively. The *sopA* (green box) and *sopB* (blue box) genes are expressed from the P*sop* promoter. The partition site *sopC* is located downstream of the *sopAB* genes. The *sopC* gene sequence is represented by a black line. The inset shows the repetitive stretch of twelve 43 bp repeats. The respective NTPases in Type-Ib (ParF), Type-II (ParM) and Type-III (TubZ) are shaded in green, and their corresponding centromere binding proteins (CBPs; ParG, ParR and TubR) are shaded in blue. The orange arrows represent transcriptional repression, whereas the yellow arrows point to centromere binding by the CBPs. **(B)** The conserved domains of F plasmid partitioning ParA_F_ protein. The N-terminal (shaded pale green), C-terminal (shaded gray) and the Walker A motifs of ParA_F_ have been represented. The Walker motifs have been highlighted in blue, and the function of each domain has been represented in orange boxes below. The amino acid residue numbers correspond to ParA_F_.

Type-Ia/large ParA proteins (~300–450 amino acids) have an extended N-terminal helix-turn-helix (HTH) motif and can act as repressors of their gene expression (Figures 1A,B) (Abeles et al., 1985; Hirano et al., 1998; Libante et al., 2001). The HTH domains help in this sequence-specific DNA binding to operator regions near their promoters (Mori et al., 1989; Davis et al., 1992; Hayes et al., 1994; Ravin et al., 2003). Type-Ia is one of the first identified DNA segregation systems and is typically encoded by partitioning loci carried in plasmids (Austin and Abeles, 1983; Ogura and Hiraga, 1983; Mori et al., 1986). Specific examples of Type-Ia include the *parABS* and the *sopABC* (*parABS*_*F*_) system of P1 bacteriophage and F plasmid, respectively. In the case of F plasmid (the one encoding for Fertility factor), the genes appear in the order of *sopA, sopB*, and *sopC*, hereafter referred to as *parA*_*F*_, *parB*_*F*_, and *parS*_*F*_, respectively. The expression of ParA_F_ and ParB_F_ are driven by a single promoter located upstream of *parA*_*F*_ (Mori et al., 1986, 1989; Hirano et al., 1998). The *parABS*_*F*_ cluster serves as the partitioning system, wherein *parS*_*F*_ serves as the centromeric sequence and comprises twelve 43-bp base pair repeats (Figure 1A). Each 43-bp sequence contains a short 16-bp inverted repeat to which ParB_F_ binds as a dimer (Hayakawa et al., 1985; Lane et al., 1987; Mori et al., 1989; Hanai et al., 1996). This ParB_F_-*parS*_*F*_ complex is then recruited to ParA_F_, which is the NTPase and functions as the motor protein (Ogura and Hiraga, 1983).

On the contrary, members of the Type-Ib subfamily are smaller (~200–250 amino acids) and lack the N-terminal HTH domain. They are thus referred to as smaller ParAs and are found in the chromosomal loci of many bacteria. However, they are also found in a few plasmids, like in TP228 of *Salmonella newport*. The ParA ATPases in the Type-Ib systems do not have any repressor functions due to the lack of the N-terminal HTH domain required for site-specific DNA binding at their promoter regions (Figure 1A). Examples of the Type-Ib system on plasmids include *parFGH* loci on the *S. newport* plasmid TP228 and the *parABS* loci (δ/*parA*, ω/*parB* and *parS*) in the plasmid pSM19035 carried by *Streptococcus pyrogenes* (de la Hoz et al., 2004; Fothergill et al., 2005; Pratto et al., 2008). Type-Ib also includes ParA from *Caulobacter crescentus* (Mohl and Gober, 1997) and Soj from *Bacillus subtilis* (ParA_Bsu_/BsSoj) (Leonard et al., 2005a; Lee and Grossman, 2006) and *Helicobacter pylori* (ParA_Hpy_/HpSoj) (Lee et al., 2006) encoded by bacterial genomes. ParB members of the Type-Ib family carry an N-terminal protein-protein interaction domain, central HTH domain and a self-dimerization domain at the C-terminus. Further, residues near the N-terminus of ParB specify their interactions with the cognate ParA (Funnell, 2016; Kawalek et al., 2020). Similar to the Type-1a system, ParB binds to *parS* and interacts with ParA, thus linking the plasmid to the motor protein ParA.

The ParA proteins, both the larger Type-1a and the smaller Type-Ib, also bind DNA in a non-sequence specific manner and is critical for their DNA segregation functions (Ebersbach and Gerdes, 2005; Leonard et al., 2005a; Hatano et al., 2007; Hester and Lutkenhaus, 2007; Castaing et al., 2008; Vecchiarelli et al., 2010; Roberts et al., 2012; Le Gall et al., 2016). The currently accepted models for DNA segregation by ParA, i.e., the diffusion-ratchet, DNA-relay and DNA hitch-hiking mechanisms (described in detail below), emphasize the importance of bacterial nucleoids. Briefly, the DNA hitch-hiking model proposes that the ParA-ATP dimers localize on the HDRs of the nucleoid to which the ParB-*parS* complex binds and stimulates the ATPase activity of ParA. The hydrolysis event converts the nucleoid bound ParA-ATP into ParA-ADP, resulting in the release of ParA from the nucleoid. This creates a zone of depletion of DNA bound ParA, and the released ParB-*parS* complex undergoes diffusive motion until captured by ParA-ATP dimers within the HDRs, resulting in a directional motion toward the highest concentration of DNA bound ParA-ATP in the cell (Vecchiarelli et al., 2012; Le Gall et al., 2016; McLeod et al., 2017). The bacterial nucleoid thus forms a key substrate for the ParA in its function as a motor protein in the equipartitioning of plasmids and chromosomes into daughter cells (Vecchiarelli et al., 2012).

### Type-II/Actin-Like ATPases

Type-II partitioning system was first discovered in the resistance plasmid R1 (Jensen and Gerdes, 1997). The partitioning system in this group contains an actin-like ATPase called ParM, an adaptor protein, ParR and a centromere site, *parC*. Monomeric ParM has an actin-like fold, with its crystal structures bearing a close resemblance to the eukaryotic actin (Bork et al., 1992; van den Ent et al., 2002). A search of the microbial genomes revealed as many as 40 different families of actin-like proteins, mostly found on plasmids (Derman et al., 2009). A few have been shown to assemble into polymeric structures and filaments with varying architectures (Becker et al., 2006; Derman et al., 2009; Popp et al., 2012; Jiang et al., 2016; Koh et al., 2019). However, the ParMRC system is the most extensively studied among the DNA partitioning systems. ParM, like actin, assembles into a two-stranded helix but with an opposite twist. While actin forms a right-handed filament, ParM helices are mainly left-handed, with 12 subunits per turn as opposed to 13 monomers in actin (Orlova et al., 2007; Popp et al., 2008; Gayathri et al., 2012). Interestingly, in the ParR (C-terminal 17 residue peptide) bound form, domains IA and IIB show a rotation of 9.1° and 10.7° toward the nucleotide, reminiscent of the shift in actin structure from G-actin to F-actin. Further, the ParR peptide binding site overlaps with the ParM polymerisation interface and thus allows ParR to bind only to the barbed end or the polymerizing end (Gayathri et al., 2012). Also, ParM grows bidirectionally with similar monomer addition rates at both ends and exhibits dynamic instability, a characteristic feature of microtubules (Gerdes et al., 2000; Garner et al., 2004).

Moreover, in the presence of a non-hydrolysable analog of ATP, these *in vitro* filaments were longer suggesting that the growth and retraction of filaments were driven by ATP hydrolysis (Garner et al., 2007). Although ParM can bind both ATP and GTP, the most preferred substrate is ATP, for which ParM has a 10-fold higher affinity than GTP (Popp et al., 2008; Galkin et al., 2009). Cryo-electron microscopy of cells overexpressing ParM has shown that the protein assembles into closely packed filament bundles with an average of 3–5 ParM filaments per bundle (Salje et al., 2009). Further studies suggest that the filaments grow by insertional polymerisation, wherein new ParM subunits are inserted at the interface of the filaments with the ParR-*parC* complex (Møller-Jensen et al., 2003; Garner et al., 2004, 2007; Salje et al., 2010; Gayathri et al., 2012). *In vitro* reconstitution of the ParMRC segregation machinery using polystyrene beads for immobilizing *parC*, purified ParR and ParM proteins, and ATP resulted in the assembly of dynamic filaments of ParM. Surprisingly, the ParM filaments were seen to grow and shrink in a manner that was reminiscent of the dynamic instability of microtubules (Garner et al., 2004, 2007). However, in the presence of another *parC* coated bead in proximity, the filaments appeared to bundle and push the beads further away, consistent with the bipolar antiparallel filament structure (Gayathri et al., 2012, 2013). Thus, ParMRC is a minimalistic tripartite system that is sufficient for plasmid segregation and does not require any additional host factors (Garner et al., 2007). Based on cryo-electron microscopy, *in vitro* reconstitution experiments, and fluorescence imaging, a “search, capture, and push” model for plasmid segregation by ParM and other actin-like proteins has been proposed (Campbell and Mullins, 2007; Garner et al., 2007; Salje et al., 2009, 2010; Gerdes et al., 2010; Gayathri et al., 2012).

### Type-III/Tubulin-Like GTPases

Partitioning machinery of this kind has been described in the pXO1 plasmid of *B. anthracis* and *B. cereus* (Tinsley and Khan, 2006; Hoshino and Hayashi, 2012), pBToxis plasmid of *B. thuringiensis* (Tang et al., 2006; Larsen et al., 2007) and more recently in bacteriophages c-st of *Clostridium botulinum* (Oliva et al., 2012) and 201φ2-1 of Pseudomonas chlororaphis (Kraemer et al., 2012). The segregation machinery comprises three components (*tubZRC*) similar to those found in Type-I and Type-II systems. However, unlike the others, the motor NTPase TubZ or PhuZ (for Phage Tubulin/FtsZ) belongs to the tubulin/FtsZ superfamily of cytoskeletal proteins (Tinsley and Khan, 2006; Larsen et al., 2007; Anand et al., 2008; Oliva et al., 2012). While, in general, the sequence similarity with eukaryotic tubulin amounts to even <15%, TubZ exhibits striking structural similarity with that of the bacterial cell division protein FtsZ and tubulin (Löwe and Amos, 1998; Nogales et al., 1998; Larsen et al., 2007; Aylett et al., 2010). TubZ undergoes polymerisation upon binding GTP (Anand et al., 2008; Chen and Erickson, 2008; Hoshino and Hayashi, 2012) and assembles into two or four-stranded filaments (Aylett et al., 2010; Montabana and Agard, 2014). Interestingly, the subunit interactions upon polymerisation leading to the coupling of GTP hydrolysis are closer to that of α/β-tubulin. Further, the protofilaments in the presence of GTPγS show a right-handed twist with 14 subunits over one complete turn (360°) and assemble into a parallel double-helical filament when expressed in *E. coli* as well (Aylett et al., 2010). The filaments undergo treadmilling, i.e., it undergoes directional polymerisation, where one end exhibits growth and the other shrinkage, which assists in DNA partitioning (Larsen et al., 2007; Aylett et al., 2010). The Type-III system is thus an example of the involvement of tubulin-like proteins in the DNA segregation in bacteria. TubR constitutes the CBP in this Type-III system that binds to the centromeric sequence *tubC* and recruits TubZ (Tang et al., 2007; Ni et al., 2010). The structure of the TubR-*tubC* complex shows that TubR binds *tubC* through an N-terminal winged helix-turn-helix motif and assembles into a DNA-nucleoprotein complex *in vitro*, forming a slight right-handed superhelix (Ni et al., 2010; Aylett and Löwe, 2012). Further, the TubR-*tubC* complex stabilizes the TubZ polymers and possibly exerts its effect by preventing the depolymerisation of TubZ filaments (Aylett and Löwe, 2012; Oliva et al., 2012). Reconstitution experiments reveal that the TubR-*tubC* complex tracks the depolymerizing minus-end of the TubZ filaments (Fink and Löwe, 2015). The bacteriophage tubulin-like protein PhuZ (TubZ_Φ*KZ*_), on the other hand, resembles the eukaryotic microtubule in many aspects. It was the first prokaryotic tubulin that was shown to exhibit dynamic instability, a property dependent upon the energy derived from GTP hydrolysis (Kraemer et al., 2012; Aylett et al., 2013; Erb et al., 2014). However, unlike tubulin, it assembles into a triple-stranded helical filament (Zehr et al., 2014). One end of the PhuZ filaments is anchored to the cell poles, and they further assemble into bipolar spindle-like structures. These bipolar spindles of PhuZ resemble eukaryotic microtubules during mitosis and help place the viral nuclei at mid-cell and control viral reproduction (Erb et al., 2014).

## The *sopABC* or *parABS* Locus in Type-I Plasmid Segregation Systems

### ParA—A Walker a Cytoskeletal ATPase

ParA protein function in Type-I partitioning systems is essential for DNA segregation of the plasmids and the bacterial chromosome, as described above. ParA belongs to the Walker A type Cytoskeletal ATPases (WACA) family of proteins and contains a Walker A motif, a Walker A' motif, a Walker B motif and a ParA specific sequence (Walker et al., 1982; Motallebi-Veshareh et al., 1990; Koonin, 1993; Lutkenhaus, 2012) (Figure 1B). As described above, the larger ParA found in Type-Ia systems has an auto-regulatory function and thus directly regulates the transcription of the *parAB* operon from its promoter (*P*_*par*_) and control levels of both ParA and ParB proteins (Mori et al., 1989; Davis et al., 1992; Hayes et al., 1994; Davey and Funnell, 1997; Hirano et al., 1998; Ravin et al., 2003; Komai et al., 2011). ParA exists in a monomer-dimer equilibrium in the cell wherein only the ATP-bound ParA dimers associate with the nucleoid, and ADP-bound dimers (and monomers) are free (Vecchiarelli et al., 2010, 2013a; Havey et al., 2012). The conformational change involves nucleotide-binding wherein the ATP bound form is dimeric (Schumacher et al., 2012; Vecchiarelli et al., 2013a). ParA exhibits a weak ATPase activity similar to the other members of the WACA superfamily (Davis et al., 1992; Watanabe et al., 1992; Libante et al., 2001; Leonard et al., 2005a; Barillà et al., 2007; Havey et al., 2012) and ATP hydrolysis activity can be stimulated 3-fold by ParB, and 1.5 fold by DNA. However, the DNA-ParB complex can exert a much stronger effect and stimulate the ATPase activity of ParA by 10–15-fold (Davis et al., 1992; Watanabe et al., 1992; Bouet et al., 2007; Ah-Seng et al., 2009). Moreover, mutants defective in ATPase activity, like ParA_P1_ K122Q and ParF_TP228_ D111A, or the mutants displaying hyper-ATPase activity, ParF_TP228_ P104A, R169A, and G179A, are all impaired in plasmid stability. These studies highlight the crucial role played by ATP hydrolysis of ParA in plasmid segregation (Fung et al., 2001; Libante et al., 2001; Dobruk-Serkowska et al., 2012; Vecchiarelli et al., 2013a; McLeod et al., 2017; Caccamo et al., 2020). Further, ParA-ATP dimers themselves have been postulated to exist in two different states/conformations, an active conformation (ParA-ATP^*^) that binds to non-specific DNA and an inactive state (ParA-ATP) that cannot bind DNA. Thus, it is the active state ATP-bound dimer (ParA-ATP^*^)_2_ that associates with the bacterial nucleoid (Vecchiarelli et al., 2010, 2013a). The ParA-ADP dimers produced soon after ATP hydrolysis can no longer associate with the bacterial nucleoid. ParA can rebind DNA only upon nucleotide exchange with ATP or reassociation with ATP and the conformational change to the ParA-ATP^*^ state. However, ParA-ADP can act as a transcriptional repressor of the ParA promoter (Davey and Funnell, 1997; Bouet and Funnell, 1999; Libante et al., 2001; Hao and Yarmolinsky, 2002; Baxter et al., 2020).

ParA has a weak auto-repression activity, and its full repressor function depends on the co-repressor ParB, together with which it strongly represses transcription of its promoter *P*_*par*_ (Friedman and Austin, 1988; Hayes et al., 1994; Libante et al., 2001). Using surface-plasmon-resonance, Bouet and group have revealed that three ParA_F_ dimers, i.e., a trimer of dimers, bind to the promoter region to suppress gene expression (Boudsocq et al., 2021). The ParB-*parS* (SopA-*sopC*) complex further enhances this auto-regulatory function. It was initially thought that only ParA-ATP and ParA-ADP, but not ParA-ATP^*^ states, were competent in binding to the operator sites *parOP* at the promoter (Davey and Funnell, 1997; Fung et al., 2001; Ravin et al., 2003; Vecchiarelli et al., 2013a). However, recent studies using the non-specific DNA binding mutant ParA_P1_ R351A suggest that ParA-ATP^*^ state is also proficient in binding *parOP*. The abrogated ns-DNA binding results in a free pool of excess ParA-ATP^*^ dimers, resulting in repression of transcription from *parOP*. This auto-repression activity of ParA thus can solely be attributed to its specific DNA binding activity mediated by the HTH domain of the protein (Baxter et al., 2020).

The active state ATP-bound dimeric conformation (ParA-ATP^*^)_2_, enables the binding of ParA molecules to the nucleoid (Vecchiarelli et al., 2010, 2013a). ParA has an affinity for DNA in a non-sequence-specific manner in this conformation. Since bacterial cells have a lot of nsDNA in the form of the nucleoid, ParA molecules are found localized to the nucleoid. Work from laboratories around the world on several ParA family proteins has shown that ParA is predominantly nucleoid bound, and its nucleoid binding function is essential for plasmid maintenance (Leonard et al., 2005a; Hayes and Barillà, 2006; Hatano et al., 2007; Hester and Lutkenhaus, 2007; Castaing et al., 2008; Vecchiarelli et al., 2010; Roberts et al., 2012; Lim et al., 2014; Volante and Alonso, 2015; Le Gall et al., 2016; McLeod et al., 2017; Caccamo et al., 2020). The interaction of ParA with nsDNA has been probed *in vitro* by several studies and visualized *in vivo* by using sophisticated fluorescence microscopy techniques (Lim et al., 2005, 2014; Hatano et al., 2007; Castaing et al., 2008; Hatano and Niki, 2010; Roberts et al., 2012; Le Gall et al., 2016; McLeod et al., 2017). Further, nsDNA binding defective mutants, ParA_F_ K340A and ParA_P1_ R351A have segregation defects suggestive of the critical role of ns-DNA binding in the process of plasmid maintenance (Castaing et al., 2008; Baxter et al., 2020). In addition, fluorescence microscopy and time-lapse imaging of ParA_F_ have suggested that the protein assembles into helical polymeric structures that undergo oscillatory behavior on the nucleoid. These observations had earlier led to suggestions that the polymerisation dynamics of ParA drove plasmid segregation (Barillà et al., 2005; Lim et al., 2005; Hatano et al., 2007; Ringgaard et al., 2009). However, as described in detail below, more recent studies on ParA dynamics *in vivo* and *in vitro* biochemical assays have favored a polymerisation-independent mechanism of DNA partitioning by ParA.

### ParB—Centromere Binding Protein or the Adaptor Protein

**Figure 2:**
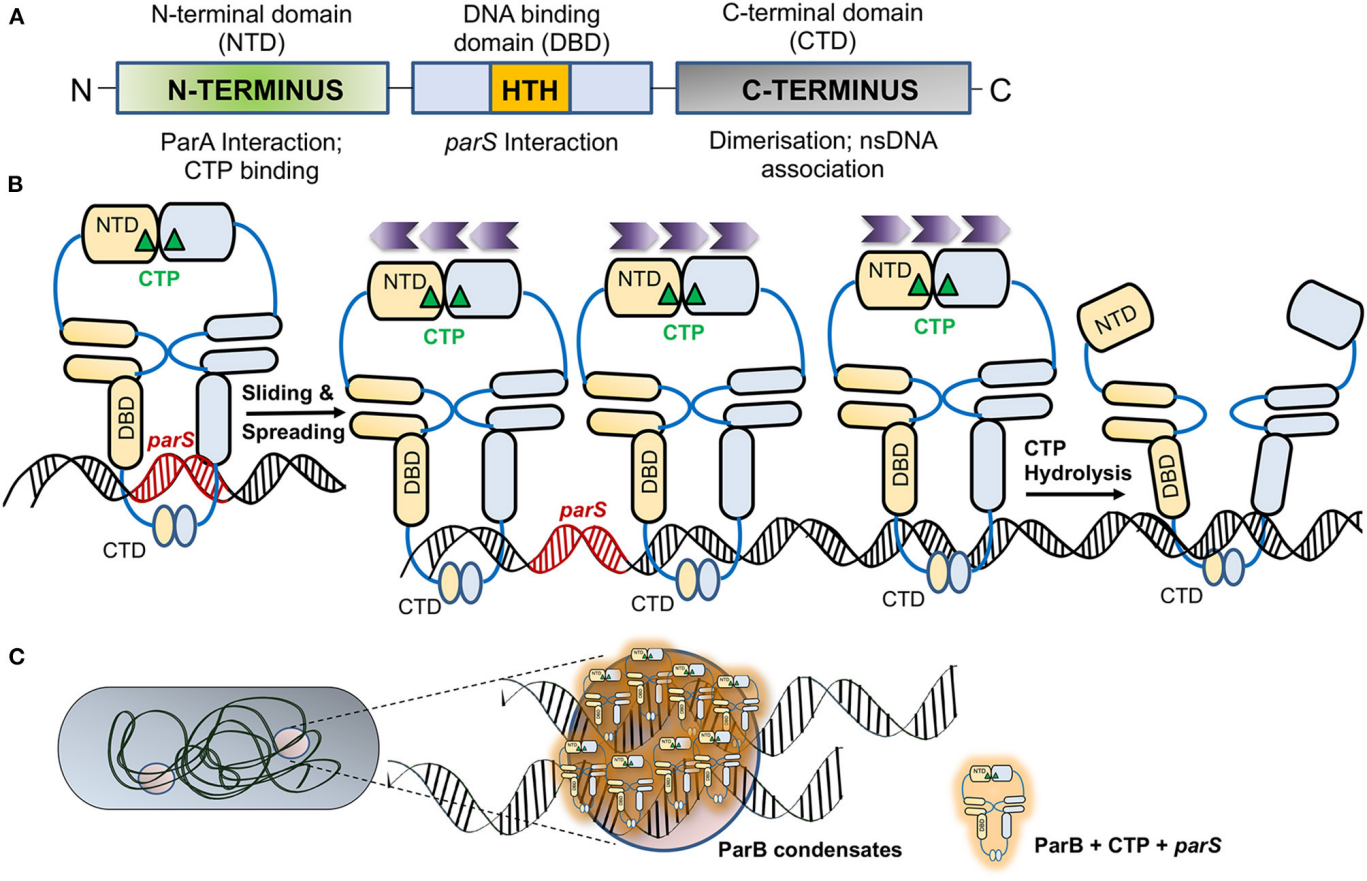
ParB—an adaptor protein and regulation of its activity by CTP. **(A)** The domain organization of ParB. The schematic representation of the ParB domain organization indicates the NTD (N-terminal domain) marked by a green box, the central DNA binding domain (DBD; marked by a blue box) contains a helix-turn-helix (HTH) motif that allows ParB nucleation on *parS* sites and is represented by an orange box. The C-terminal domain (CTD) responsible for dimerization is shaded gray. The function of each domain is shown below. **(B)** Association of ParB with CTP. Upon association of apo-ParB NTD with CTP (marked by a green triangle), the ParB molecules form homodimers and associate with *parS* (indicated in red). The ParB-CTP dimers clamped to the DNA further undergo sliding and spread across the *parS* proximal sites. Sliding and spreading of ParB across DNA may not require CTP hydrolysis. The direction of spreading is indicated by the purple chevrons (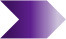). However, upon CTP hydrolysis, the NTDs of two ParB are disassociated, leading to a switch from dimer to apo-form, leading to the release of ParB from the DNA. **(C)** Liquid-Liquid Phase Separated (LLPS) condensates formed by ParB. The schematic depicts a bacterial cell with ParB condensates, and the zoomed inset shows several ParB-CTP dimers that associate *parS* sites containing DNA to form the condensates.

ParB is a DNA binding protein that binds to specific repeat sequences found in plasmids or genomes (*parS*) and forms an active component of the bacterial DNA segregation machinery. The role of chromosomally encoded ParB in various cellular functions and DNA partitioning has been recently reviewed in detail by Kawalek et al. (2020). The crystal structure of the full-length ParB protein from *B. subtilis* (21–218 aa), also known as SpoOJ, provides details about its domain organization (Soh et al., 2019). ParB comprises of different domains: the N-terminal domain, a central DNA binding HTH motif and a C-terminal domain, all connected by flexible linkers (Funnell, 1991, 2016; Schumacher and Funnell, 2005; Badrinarayanan et al., 2015; Soh et al., 2019) (Figure 2A). The C-terminal domain plays a pivotal role in the homo-dimerization of ParB (Khare et al., 2004; Leonard et al., 2005a). Mutation R149G within the HTH motif affects *parS* binding (Autret et al., 2001; Gruber and Errington, 2009; Fisher et al., 2017) and the central DNA binding domain thus is critical for the recognition and binding of ParB to *parS* sites (Leonard et al., 2005a; Schumacher and Funnell, 2005). The DNA binding domain or HTH motif plays a vital role in specific DNA interaction with *parS* sites and spreading to the adjacent DNA after binding to the *parS* sites. Spreading is a crucial feature of ParB, and it involves the formation of higher-ordered complexes. ParB is known to initiate binding to DNA at *parS* sites and spread over *parS* flanking regions, often covering a large span of the nsDNA (reviewed in Jalal and Le, 2020; Kawalek et al., 2020). The N-terminal stretch is necessary for protein oligomerisation as well as interaction with ParA. It is defined by a conserved stretch of Arginine residues GERRxRA, referred to as the arginine patch. Mutations within the arginine patch impair ParB spreading as well as nucleoid segregation functions (Yamaichi and Niki, 2000; Ah-Seng et al., 2013; Chen et al., 2015). This patch is thus essential for the spreading of ParB, foci formation and DNA partitioning (Rodionov et al., 1999; Autret et al., 2001; Bartosik et al., 2004; Breier and Grossman, 2007; Kusiak et al., 2011; Graham et al., 2014; Funnell, 2016). Further, ParB is thought to stimulate the ATPase activity of ParA *via* an arginine finger (R-finger) motif contained within the N-terminal region (Ah-Seng et al., 2009; Zhang and Schumacher, 2017).

### ParB Activity Is Regulated by CTP Binding and Hydrolysis

Recent structural and biochemical studies on ParB have revealed that ParB is a CTPase with the arginine patch forming the catalytic center (Soh et al., 2019; Jalal et al., 2020; Osorio-Valeriano et al., 2021). This surprising finding was revealed by crystal structures of ParB of *B. subtilis* and PadC, a ParB member from *M. xanthus*, which were found to be associated with CDP and CTP, respectively (Soh et al., 2019; Osorio-Valeriano et al., 2021). The revelation of these ParB structures with CTP as a cofactor has opened up new avenues of research and questions on the role of CTP in regulating the process of ParB spreading and DNA partitioning (Jalal et al., 2020, 2021). CTP binding results in dimerization of the N-terminal domains of two ParBs in the presence of Mg^2+^, which acts as a cofactor in the process. Such self-dimerization is sufficient to cause the formation of a clamp that entraps the DNA (Soh et al., 2019; Osorio-Valeriano et al., 2021). Recent studies using optical tweezers and microscopy have shown that binding of CTP or the non-hydrolysable analog CTPγS enhances ParB binding to *parS* sites and results in DNA condensation (Balaguer et al., 2021). A conformational change mediates ParB dimerization upon binding to CTP and *parS*. Upon CTP hydrolysis, CDP has a low affinity for ParB, and thus, it dissociates from the NBD of ParB and results in a switch to its apo-form (Figure 2B). Moreover, studies have shown that ParB networks show a continuous exchange and turnover of ParB at both protein-protein and protein-DNA interfaces (Madariaga-Marcos et al., 2019). Consistently, CTP hydrolysis defective mutants, Q52A and E93A in *M. xanthus* ParB, exhibit abnormal chromosome segregation (Osorio-Valeriano et al., 2021). Further, ParB spreading to distal regions from *parS* sites could be prevented by the presence of protein roadblocks, suggesting that ParB spreading on nsDNA was one-dimensional. CTP binding thus plays an essential role in ParB spreading and DNA condensation (Balaguer et al., 2021).

ParB has been recently shown to undergo Liquid-liquid phase separation (LLPS), favoring condensate formation in the presence of CTP (Babl et al., 2022). Liquid-liquid phase separation is a thermodynamic process wherein a homogeneous mixture of two or more molecules de-mixes into distinct phases and thus helps compartmentalize membrane-less organelles (Hyman et al., 2014; Guilhas et al., 2020; Babl et al., 2022). In most eukaryotic subcellular structures, like germline P-bodies (Brangwynne et al., 2009), stress granules (Protter and Parker, 2016) etc., compartmentalization depends upon LLPS. In bacteria, LLPS has been implicated in the assembly of carboxysomes and divisome protein FtsZ (Monterroso et al., 2019; Wang et al., 2019; MacCready et al., 2020). Recently, ParB has also been shown to exist in two different phases, a gas phase and a liquid phase. Further, in a process similar to the LLPS in eukaryotic proteins, ParB from *Corynebacterium glutamicum* also forms spherical assemblies in the presence of crowding agents, is affected by alterations in ionic strength and is stabilized by potassium glutamate (Babl et al., 2022). The condensation of ParB is further favored by the presence of *parS* containing DNA and the motor protein ParA helps in the separation of these condensates (Figure 2C). Moreover, in the case of the ParA_F_ K120Q mutant, the condensates merge and do not segregate suggesting that the ParB stimulated ATPase activity of ParA is required to separate these condensates (Guilhas et al., 2020; Babl et al., 2022).

## The Mechanism and Models of Dna Partitioning by ParA

**Figure 3:**
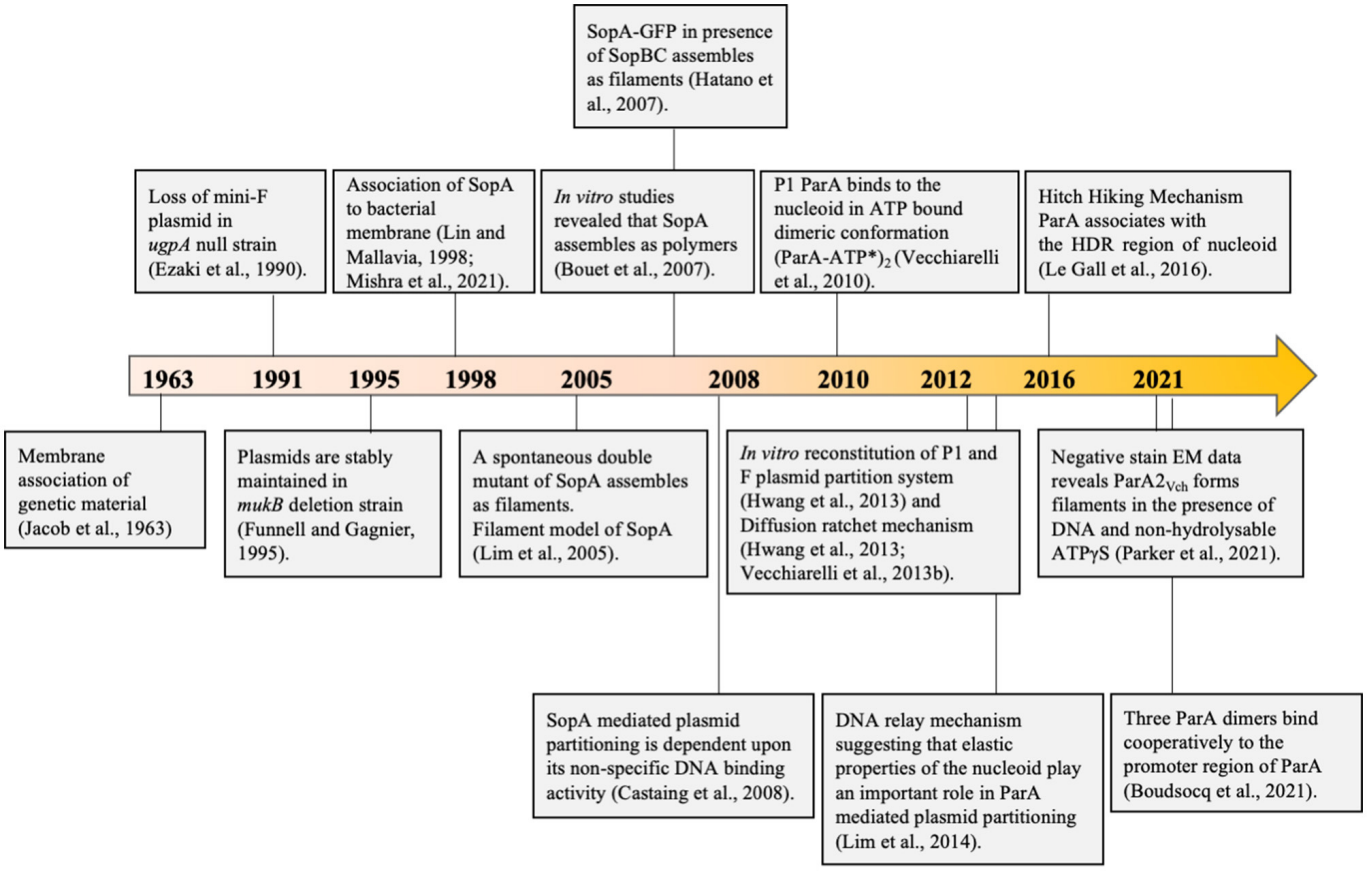
A timeline depicting important milestones in our understanding of the models proposed for the mechanism of DNA segregation. In 1963, Francois Jacob proposed the membrane tethering model wherein the chromosome was believed to be associated with the bacterial membrane. The chromosomes would be pulled along as the cell grows and eventually separated. This was further supported by Ezaki et al. (1991) and Lin and Mallavia (1998) suggesting that the DNA was probably linked to the membrane through the partitioning protein ParA_F_ (SopA). Toward the early part of the twenty-first century, with the emerging concept of the cytoskeleton in bacteria, the first polymerisation based filament models of segregation replaced the erstwhile membrane model, wherein it was proposed that the plasmid partitioning protein ParA polymerizes to push apart the plasmids to the two opposite ends of the cell. This model was supported by the findings of Lim et al. (2005), Bouet et al. (2007), and Hatano et al. (2007). Recent findings by Parker et al. (2021) suggest oligomerisation on DNA. Castaing et al. (2008) reported the association of the ParA with the nucleoid and, together with several other findings from many labs across the globe, led to abandoning the popular polymerisation model and models accounting for nucleoid association like the diffusion-ratchet mechanism and Hitch-Hiking mechanism were put forth. These form the current models and are the most accepted models of plasmid and chromosome segregation.

**Figure 4:**
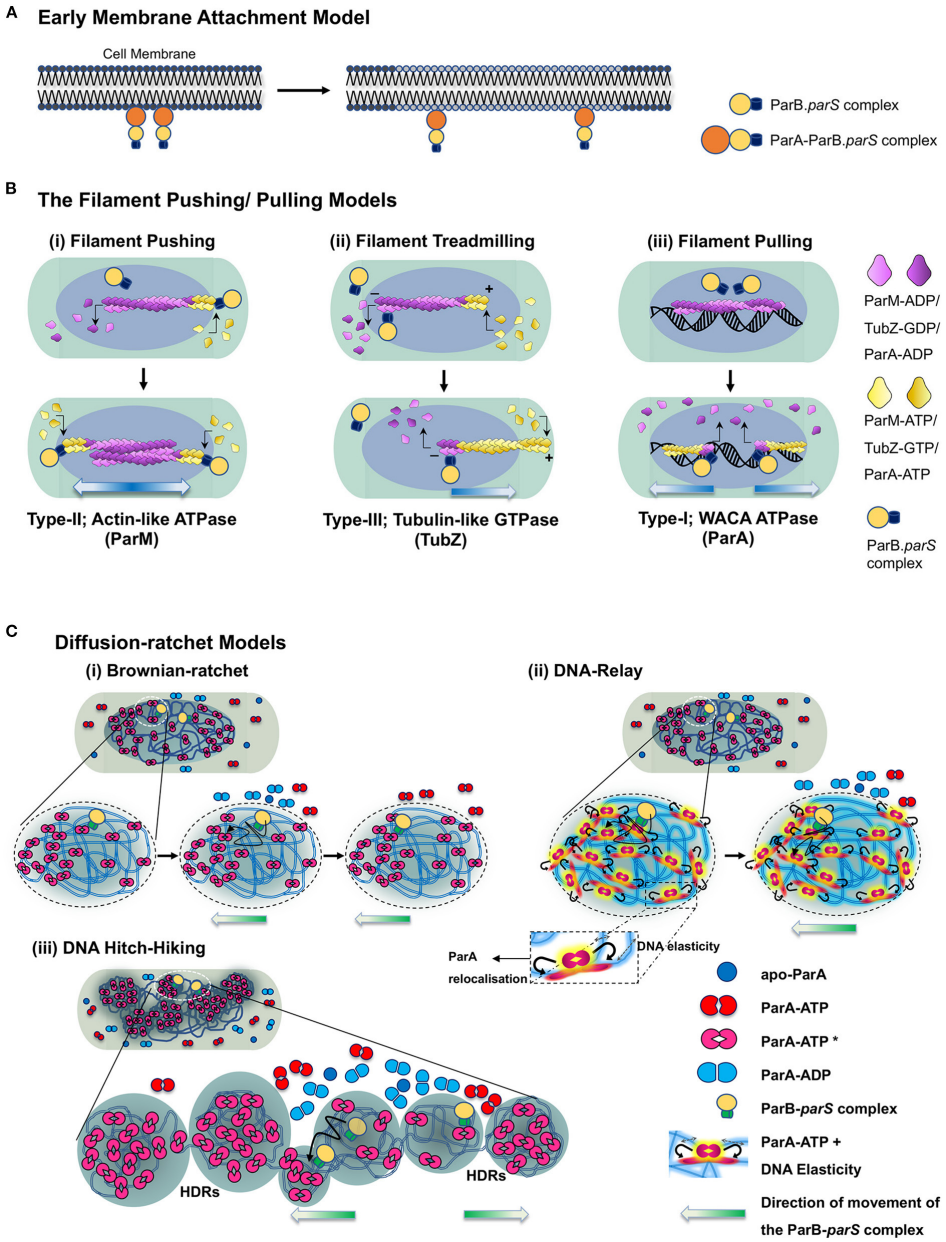
Model depicting the mechanisms of ParA proteins in DNA partitioning. **(A)** Early membrane attachment model. An early model for DNA partitioning had suggested membrane tethering of the genetic material. As per this model, DNA is associated with the membrane, and cell elongation pulls the replicated DNA apart, which physically separates into the newly formed daughter cells upon cell division. It was originally proposed for chromosomal DNA and later proposed for F plasmids as well. ParA_F_ (SopA) was suggested to be the membrane tether for the plasmids. **(B)** Filament pushing/pulling model. A filament model invokes the formation of long-range polymeric structures (μm scale). The filament polymerisation mechanisms are utilized by Type-II and Type-III systems. In the case of the Type-II system, the polymerisation of an actin-like ATPase (ParM) generates the pushing forces to segregate the plasmids, whereas treadmilling of the polymers (TubZ) drives the plasmids to cell poles in the Type-III system. Although a filament model in which the plasmids are pulled to the cell poles has been proposed for ParA (Type-I) as well, the existence of continuous filaments of ParA *in vivo* is debatable. **(C)** Diffusion-ratchet models. The diffusion-ratchet models posit that a chemophoretic gradient formed by ParA-ATP molecules bound to the nucleoid drives the movement of the plasmid bound partitioning complex up the gradient. The ParB-*parS* partition complex, on encountering DNA bound ParA-ATP dimers (ParA-ATP* state), stimulates the ATP hydrolysis of ParA, converting it into ParA-ADP and can no longer be associated with the nucleoid. The released ParB-*parS* complex undergoes Brownian motion but is constrained by the cell membrane and rebinds the nucleoid-associated ParA-ATP complex in its vicinity. Cycles of such release, diffusion and capture allow the partition complex to move up the ParA-ATP gradient on the nucleoid to achieve a unidirectional motion. (i) In a simplistic Brownian diffusion and capture of the partition complex (ParB-*parS*) by ParA-ATP dimers on the surface of the nucleoid, the plasmid surfs on the nucleoid and migrates toward the cell poles. (ii) In the DNA-relay models, the elasticity of the chromosomal DNA (indicated by haze around the DNA and double arrows in the inset) supports the movement of the partition complex, and simple diffusion by Brownian motion alone is not sufficient to achieve the long-distance directional motion observed in cells. Both the above models imply that the movement of the partition complex occurs on the surface of the nucleoid. (iii) The DNA Hitch-Hiking model instead suggests that the DNA segregation complex undergoes diffusive movement deep within the chromosomes in the High-density regions (HDRs). The direction of movement of the partitioning complex is indicated by gradient arrows (blue in the case of filament models and green in the case of diffusion-ratchet models).

François Jacob put forth the first DNA segregation and separation model in bacteria. The bacterial inner membrane and cell growth were proposed to play a central role in pulling the chromosome apart during cell division. This model was primarily derived from electron microscopy of bacterial cells showing tethering of the genetic material to the bacterial inner membrane. As per this model, replicated DNA becomes tethered to the cytoplasmic membrane. As the cell elongates, the chromosomes are pulled apart to the two opposite poles of the cells, following which cell division ensues and separates the replicated DNA (Jacob et al., 1963). This mode of DNA segregation was also assumed to be true for F plasmids. A timeline depicting the evolution of our understanding of DNA segregation in bacteria is shown in Figure 3. The Jacob model was further supported by the findings that the plasmid partitioning proteins ParA_F_ of F plasmid (FSopA) and that of Q plasmid (ParA_Q_/QSopA) of *Coxiella burnetii* associate with the cell membranes (Lin and Mallavia, 1998). The study involved biochemical membrane fractionation, floatation assays and immunoelectron microscopy, which suggested that a fraction of the respective ParA proteins were localized to the bacterial inner membranes. Further, phosphatase assays using the periplasmic PhoA protein indicated that the N-terminal residues in ParA_F_ and ParA_Q_ might specify membrane association (Lin and Mallavia, 1998). Consistent with the ideas around the time, these studies resulted in a model being proposed for plasmid partitioning, wherein the plasmid-ParB complex became associated with the membrane *via* ParA, and DNA partitioning was driven by cell elongation (Figure 4A). Plasmid partitioning *via* membrane association of ParA was further supported by beautiful genetic and plasmid localization studies showing the abundance of F plasmids in anucleate cells (Ezaki et al., 1991), although early studies using *mukB* indicated a general role for the nucleoid as well (Niki et al., 1991). More recently, we have identified a potential amphipathic helix in the C-terminus of ParA_F_ (Mishra et al., 2021). However, the sufficiency of the C-terminal helix to bind bacterial membranes has not been examined.

However, further studies that directly visualized F plasmid and other partitioning proteins in bacteria using fluorescence microscopy and imaging techniques never revealed any membrane localization for ParA. On the contrary, ParA was predominantly associated with chromosomal DNA and localized on the bacterial nucleoid. At the turn of the millennium, as the concept of bacterial cytoskeleton had just emerged, a cytoskeletal filament model in the year 2005 was proposed (Figure 4B). The cytoskeletal model was based on the observations that ParA_F_ and ParF (ParA_TP228_) assembled into polymeric structures (Barillà et al., 2005; Lim et al., 2005). Pogliano and colleagues, using Nile red staining and light microscopy, found that ParA_F_ assembled into filaments *in vitro* in the presence of ATP and grew at a rate of ~0.18 ± 0.05 μm per minute (Lim et al., 2005). The polymerisation of ParA_F_ was further ascertained by *in vivo* fluorescence imaging of functional C-terminal GFP fusions to ParA_F_ in *E. coli* (Lim et al., 2005; Hatano et al., 2007) and *in vitro* by transmission electron microscopy (Bouet et al., 2007). ParA of pB171 plasmid also exhibits similar polymeric structures in the presence of DNA (Ringgaard et al., 2009). The cytoskeletal filament model was later supported by super-resolution imaging studies of *C. crescentus* ParA (Ptacin et al., 2010). A chromosome pulling model similar to the eukaryotic burnt-bridge model for tubulin mediated chromosome segregation was proposed (Ptacin et al., 2010). As per this model, ParA undergoes polymerisation forming a filament structure that pulls the plasmid to the opposite ends of the cell *via* the ParB-*parS* complex (Ptacin et al., 2010) and contrasted with the pushing mechanism employed in the R1 plasmid by the actin-like ParM protein (described above). Further, members of the smaller ParA family, like Soj (ParA_Bsu_ and ParA_Tth_), have been reported to undergo polymerisation in the presence of ATP and DNA (Leonard et al., 2004, 2005a; Barillà et al., 2005; Pratto et al., 2008; Soberón et al., 2011; Schumacher et al., 2012; Volante and Alonso, 2015).

However, several research laboratories working on various ParA proteins from diverse bacterial genera found the polymerisation and filament-pulling model inconsistent with the emerging biochemical and cell biological evidence in the following decade. Thus, the models based on polymerisation-mediated DNA segregation by ParA proteins of the Type-I system were soon superseded by models favoring chemophoretic gradients formed by ParA on the bacterial nucleoids along the cell's long axis (Figure 4C).

An overwhelming amount of literature on ParA from several species and exhaustive biochemistry and super-resolution imaging argue against polymerisation mediated partitioning. Instead, they support a diffusion-ratchet mechanism (Hatano and Niki, 2010; Hwang et al., 2013; Vecchiarelli et al., 2013b; Hu et al., 2017; reviewed in Brooks and Hwang, 2017) or DNA-relay mechanism (Lim et al., 2014; Surovtsev et al., 2016) for the movement of plasmids or chromosomal *oriC* proximal ParB-*parS* complexes toward the cell poles. The diffusion ratchet models are principally derived from the *in vitro* reconstitution experiments which more or less replicate the *in vivo* conditions (Vecchiarelli et al., 2010, 2013b, 2015; Hwang et al., 2013; reviewed in Brooks and Hwang, 2017). Mizuuchi and the group utilized *in vitro* reconstitution experiments mimicking the minimalistic plasmid partitioning apparatus on a glass slide coated with DNA to resemble the bacterial nucleoid. They were able to observe the dynamics of the partitioning machinery and the relevance of non-specific DNA binding in the ParA mediated movement of the partitioning complex using TIRF microscopy (Hwang et al., 2013; Vecchiarelli et al., 2013b, 2014). Moreover, this was also supported by *in vivo* imaging data reported for other members of the ParA superfamily (Fogel and Waldor, 2005; Lim et al., 2005; Hatano et al., 2007; Hester and Lutkenhaus, 2007; Hatano and Niki, 2010; Le Gall et al., 2016; McLeod et al., 2017). One of the main attributes of this model was that the ParA proteins bind the non-specific DNA (nucleoid) as dimers in the presence of ATP and do not assemble into polymers or filamentous structures. DNA-relay mechanism was subsequently proposed to explain the movement and dynamics of *C. crescentus* ParA observed during the relocation of the replicated origin from the old pole to the new pole (Lim et al., 2014). The model also considered the elastic properties of the bacterial nucleoid that helps in segregating the plasmids. The initial asymmetry in ParA localization is brought about by the stochasticity in the ATP hydrolysis in the region surrounding the partition complex. This asymmetry creates an imbalance in the elastic forces experienced by the partitioning complex and results in an incremental movement of the complex in the direction opposite to the site of ATP hydrolysis. When two partition complexes are in close proximity, a large zone of depletion of ParA dimers occurs and results in an apparent repulsion and change in the direction of motion of the partition complexes (Surovtsev et al., 2016). Fluctuations in the DNA positions caused by stretching forces and the elastic dynamics of the nucleoid act to position the partitioning complex at a new location and relay it across the length of the cell (Lim et al., 2014; Surovtsev et al., 2016). The diffusion-ratchet and DNA-relay models assume the DNA partitioning process ensues on the surface of the nucleoid. Further, the diffusion-ratchet mechanism invokes confinement by the inner membrane to prevent diffusion of the free plasmid in three dimensions and limit movement to almost a 2D surface (Vecchiarelli et al., 2014). However, recent super-resolution imaging, using structured illumination (SIM) and multi-focus microscopy (MFM), of ParA_F_ and ParF_TP228_ strongly suggest that the movement of the plasmid (the ParB-*parS* complex) is not the surface but rather appears to be deep within the nucleoid space (Le Gall et al., 2016; McLeod et al., 2017). These data have led to the proposition of a DNA Hitch-Hiking mechanism, a model that is fully consistent with the diffusion-ratchet and DNA-relay mechanisms (Le Gall et al., 2016) (Figure 4C). Our current understanding of the ParA mediated DNA segregation can thus be summarized as below:

The accurate positioning and partitioning of DNA during each round of cell division begins with its replication. The active form of ParA, i.e., the ParA-ATP^*^ state, binds nsDNA as a dimer (ParA-ATP^*^)_2_ and remains associated within the high-density regions (HDR) within the bacterial nucleoid. The repetitive centromeric sequence, *parS*, in the plasmids or in the chromosome that lies proximal to the origin (*oriC*), is bound by ParB and results in clustering or spreading of ParB around *parS* sequences. This ParB-*parS* complex then hovers around the cell space of the bacterium, searching for the nucleoid bound ParA-ATP^*^ dimers. The ParB-centromere complex, upon encountering (ParA-ATP^*^)_2_, stimulates the ATPase activity of ParA. ATP hydrolysis results in the conversion of ParA-ATP^*^ to ParA-ADP, which can no longer remain bound to the bacterial nucleoid and is thus released into the cytosol. The release of ParA molecules from the nucleoid creates a zone of depletion of DNA-bound ParA-ATP in the vicinity of the ParB-centromere complex. This results in a local gradient of (ParA-ATP^*^)_2_ on the nucleoid, and the ParB-centromere complex, driven by Brownian motion, moves up the concentration gradient toward the DNA-bound ParA-ATP. The randomly chosen initial direction of motion of the ParB-*parS* complex can bias the movement in the same direction driving the long-range unidirectional movement of the plasmid. In the DNA Hitch-Hiking model, ParA-ATP molecules are bound to the nucleoid in regions of high density (HDR), and the partition complex hops from one HDR to another resulting in the progressive directional motion of the ParB-bound DNA complex.

Meanwhile, the ParA-ADP or the apo-ParA can possibly associate with ATP to form the ParA-ATP dimer [(ParA-ATP)_2_] almost instantly, given the relatively higher concentrations of ATP in the cell, which are usually in the millimolar range. However, the ParA-ATP dimer [(ParA-ATP)_2_] cannot immediately bind DNA and is slowly converted to a (ParA-ATP^*^)_2_ state, which can re-associate with the bacterial nucleoid and contribute to the gradient. This time-delay in the nucleoid association of ParA-ATP is crucial to the sustenance of the ParA-ATP gradient and the unidirectional motion of the partitioning complex in all diffusion-ratchet based models (Vecchiarelli et al., 2010, 2013a; Hu et al., 2015, 2017; Le Gall et al., 2016; Surovtsev et al., 2016; McLeod et al., 2017). Cycles of such binding and release of the ParA-ATP within the nucleoid eventually mediate displacement of the replicated plasmids away from the cell division site, ensuring equipartitioning of the genetic material.

Interestingly, recent electron microscopy studies on *V. cholerae* ParA2 and archaeal SegAB complex show that ParA2_Vch_ and SegA assemble into filaments in the presence of DNA and ATP or non-hydrolysable ATP (Hui et al., 2010; Parker et al., 2021; Yen et al., 2021). Further, electron microscopy and chromatography of purified ParA_P1_ in the presence of ATP and ns-DNA indicate the assembly of ParA_P1_ into polymeric structures at very high concentrations of the protein. While its physiological relevance is yet to be determined, they suggest that the filament structures are also likely in the ParA-ATP^*^ state (Dunham et al., 2009; Vecchiarelli et al., 2010). Although, these findings could revitalize the polymeric cytoskeleton models for ParA mediated DNA segregation, ParA2_Vch_ does not form stable polymers and is likely to bind DNA cooperatively as higher-order oligomers, which could be compatible with Brownian-ratchet models and not filament pulling models (Chodha et al., 2021).

## Walker a Cytoskeletal ATPases in Bacterial Chromosome Segregation

### Soj in *Bacillus subtilis*

The partitioning locus in the case of *B. subtilis* consists of two proteins, Soj and SpoOJ and a centromere-like sequence *parS* (Ireton et al., 1994; Leonard et al., 2005a; Lee and Grossman, 2006). While Soj belongs to the ParA family, SpoOJ is a ParB-like protein, both of which were initially isolated as sporulation genes and hence the names Soj-SpoOJ. We refer to Soj and SpoOJ proteins as ParA_Bsu_ and ParB_Bsu_, respectively. Overexpression of ParA_Bsu_ or deletion of ParB_Bsu_ leads to the formation of aberrant nucleoid morphology and anucleate cells (Lee and Grossman, 2006). ParA_Bsu_ is a smaller Type-Ib Walker A ATPase with only 21% sequence similarity to ParA_F_ protein. ParA_Bsu_ works cooperatively with ParB_Bsu_ to enable chromosome segregation. ParB_Bsu_ binds to the eight repetitive sequences on *parS* located on both sides of the origin of replication *oriC*. When visualized by fluorescence microscopy, this complex appears as discrete spots on binding ParA_Bsu_ proteins. A green fluorescent protein (GFP) fusion of ParA_Bsu_ shows nucleoid localization of the protein. However, when co-expressed with ParA_Bsu_, it leads to the formation of discrete spots that colocalise with the ParB-*parS* complex in the cell (Leonard et al., 2005a; Scholefield et al., 2011). GFP fusions of ParA_Bsu_ seem to undergo oscillatory movement (Leonard et al., 2005a), a characteristic feature of the ParA superfamily (Raskin and de Boer, 1999; Hatano et al., 2007; McLeod et al., 2017). While ParA_Bsu_ controls the condensation of ParB_Bsu_ foci, ParB_Bsu_ regulates the dynamic behavior of ParA_Bsu_, and thus both the proteins are functionally interlinked. Biochemical studies show that ATP bound ParA_Bsu_ exists as a dimer, and an ATPase mutant D44A was crystallized in dimeric form (Leonard et al., 2005a). Non-specific DNA binding and nucleoid association of ATP-bound ParA_Bsu_ is mainly mediated by surface-exposed arginine residues (Hester and Lutkenhaus, 2007). Further, electron microscopy data suggest that, like *Thermus thermophilus* ParA (ParA_Tth_/TthSoj), ParA_Bsu_ also forms a nucleoprotein filament, showing that the dimeric form of ParA_Bsu_ recruits other ParA_Bsu_ dimers facilitating the formation of a polymeric structure (Leonard et al., 2004, 2005a). Interaction with ParB_Bsu_ stimulates the ATPase activity of ParA_Bsu_, and ADP-bound forms dissociate from DNA (Scholefield et al., 2011). In addition to its role in DNA segregation, the ParA_BSu_ is also a transcriptional regulator that controls the expression of many sporulation genes, including *spo0A, spoIIA, spoIIG*, and *spoIIE* (McLeod and Spiegelman, 2005).

### MipZ and ParA in *Caulobacter crescents*—Two WACA Proteins Coordinate DNA Segregation

In *C. crescentus*, two WACA family members coordinate to promote segregation (Mohl and Gober, 1997; Thanbichler and Shapiro, 2006; Toro et al., 2008; Corrales-Guerrero et al., 2020). Both proteins can interact with the centromere binding protein, ParB, which binds to the *parS* DNA sites that lie close to the origin of replication (*oriC*) in *C. crescentus*. At the beginning of the cell cycle, *parS* sites are anchored to the cell poles by the interaction of ParB with PopZ, a polarly localized protein. However, at the onset of the S-phase, the chromosome duplicates and one copy of the ParB-*parS* complex moves to the other pole of the cell driven by the nucleoid bound ParA-ATP gradient (Thanbichler and Shapiro, 2006). MipZ, another ParA member, binds to ParB at both the cell poles and forms a concentration gradient along the cell length, with the highest concentration being on the poles and the lowest at the mid-cell site (Easter and Gober, 2002; Thanbichler and Shapiro, 2006). MipZ is an antagonist of FtsZ, and a high concentration of MipZ prevents the formation of Z-ring at the poles but promotes its assembly at the mid-cell site (Thanbichler and Shapiro, 2006; Du and Lutkenhaus, 2012). Although ParB can bind both ParA and MipZ, the roles played by ParB at the two poles are different. At the old pole, where ParB is bound to *parS* and interacts with ParA, ParB acts to stimulate the ATP hydrolysis of ParA-ATP. This stimulation of the ATPase activity of ParA by the ParB-*parS* complex aids in generating the ParA gradient for the effective movement of the ParB-*parS* complex to the new pole of the cell (Corrales-Guerrero et al., 2020).

In contrast, MipZ binds ParB at both the old and new poles; ParB plays a role of a catalyst and promotes dimerization and binding of MipZ to DNA by recruiting monomers. However, ATP hydrolysis due to the intrinsic ATPase activity of MipZ results in a release of the MipZ monomers from the nucleoids at ParB distal regions. Since ParB is localized at cell poles, the association of MipZ with the nucleoid is at its lowest concentration at the mid-cell position (Kiekebusch et al., 2012). Both MipZ and ParA have conserved residues that bind nsDNA and have nucleoid binding activities that help DNA segregation (Du and Lutkenhaus, 2012). Thus, both MipZ and ParA work synergistically in *C. crescentus* DNA segregation, and both are indispensable for accurate chromosome partitioning. Further, MipZ serves another critical function of precisely positioning the Z-ring for cell division in *C. crescentus*.

### Chromosome Segregation Machinery in Bacterial Pathogens

#### Vibrio cholerae

*V. cholerae*, the causative agent of cholera, is a gram-negative bacterium with two chromosomes, each with its *par* locus (Heidelberg et al., 2000). Although both the chromosomes encode the Type-I mechanism of segregation (Fogel and Waldor, 2005, 2006; Yamaichi et al., 2007), they employ different strategies for their transmission to daughter cells (Venkova-Canova et al., 2013). The 2.4-Mb chromosome I makes use of the Type-Ib system, while the smaller 1.6-Mb Chromosome II uses the Type-Ia segregation mechanism. Studies on the partitioning of chromosome II have indicated a role for ParA2_Vch_. Genetic studies have shown that the deletion of *parAB2* causes loss of Chromosome II from the cells, and thus *parAB2* locus seems essential for Chromosome II segregation (Yamaichi et al., 2007). Further, it has been shown that ParA2_Vch_ binds to the non-specific DNA like many other members of the superfamily, as mentioned above (Hui et al., 2010; Chodha et al., 2021). Interestingly, recent cryo-EM studies on ParA2_Vch_ have reported its assembly into a polymeric nucleoprotein complex in the presence of ATPγS and DNA (Parker et al., 2021).

#### Helicobacter pylori

While Soj in *H. pylori* is a member of the ParA superfamily of proteins and bears 22–48% sequence similarity to ParA_F_ protein, SpoOJ (HP1138 gene) is the ParB homolog. The *soj* (HP1139) and *spoOJ* (HP1138) genes, together with the two putative *parS* sites, are located within 20–30% of the origin-proximal region of the circular chromosome of *H. pylori*, as observed in other species (Lee et al., 2006; Chu et al., 2019). On binding to nucleotide, HpSoj/ParA_Hpy_ forms a dimer, a feature that has also been observed in its crystal structure. ParA_Hpy_ is a weak ATPase, and its activity is stimulated by non-specific DNA and ParB_Hpy_/HpSpoOJ, a typical trait of the ParA family of proteins. However, unlike ParA_Tth_ or ParA_Vch_, electron microscopy failed to detect any ParA_Hpy_ filaments and thus excluded the possibility of ParA polymers driving DNA segregation. Moreover, a stretch of basic residues that allows ParA_Hpy_ to bind nsDNA was identified, suggesting that a diffusion-ratchet mechanism (described below) might be operative to partition DNA (Chu et al., 2019).

#### Pseudomonas aeruginosa

*P. aeruginosa*, a γ-proteobacterium, is an opportunistic pathogen and is the causative agent of morbidity in cystic fibrosis patients (Stover et al., 2000). It encodes a *parABS* system in its genome that helps segregate its large 6.3 Mb chromosome (Lagage et al., 2016). The *par* genes are located ~8 kb from *oriC*, and the deletion of ParA or ParB leads to chromosomal segregation defects resulting in an increased production of anucleate cells (Lasocki et al., 2007). ParB of Pseudomonas has been postulated to be a NAP (Nucleoid Associated Protein) exhibiting specific interaction with *parS* sites (Kusiak et al., 2011), interaction with several hepta-nucleotide sequences in the genome and regulating transcription of various genes (Kawalek et al., 2018). Although ten 16-bp palindromic *parS* sites have been reported in the genome (Bartosik et al., 2004; Jecz et al., 2015), only four are proximal to the origin, *oriC* (Livny et al., 2007). Although, ParB binds to these *oriC* proximal four *parS* sites (Kusiak et al., 2011; Lagage et al., 2016), just one of them is sufficient for ParB binding and accurate chromosome segregation (Jecz et al., 2015). Surprisingly, displacement of *parS* from its native site does not affect chromosome segregation. The *parABS* locus remains the first locus to be segregated following replication, suggesting parS action is independent of its location in the chromosome (Lagage et al., 2016).

#### Mycobacterium tuberculosis

*M. tuberculosis* is the causative agent of tuberculosis, and its genome also encodes a parABS system that includes a parS site, parA (Rv3918) and parB (Rv3917) genes, in addition to two other parA homologs (Rv1708 *and* Rv3213). A study using Transposon site hybridization (TraSH) indicated that the parAB genes within the parABS loci are essential for growth and viability and could not be deleted in *M. tuberculosis* (Sassetti and Rubin, 2003). However, in *M. smegmatis*, ParA is not essential but is required for normal growth (Ginda et al., 2013). While all the three ParA homologs are localized at the cell poles, ParA_Mtb_Rv3918_ colocalises with ParB at either of the poles and exhibits localization at the polar or mid-cell position that overlaps with nucleoid or inter-nucleoid regions, which suggests oscillations but require further time-lapse imaging to ascertain foci movements (Maloney et al., 2009). In *M. tuberculosis*, ParB is phosphorylated by a Ser/Thr Protein Kinase (STPK) and negatively regulates ParB activity by inhibiting interaction with parS and ParA. Mutations that mimic phosphorylation affect the cellular localization of ParB_Mtb_ and lead to impaired chromosome segregation (Baronian et al., 2015). Further, many studies utilizing *M. smegmatis* as a model organism confirm the dynamic localization of the ParA and ParB-parS complexes in mycobacteria (Jakimowicz et al., 2007; Ginda et al., 2013; Santi and McKinney, 2015; Trojanowski et al., 2015; Uhía et al., 2018). Interestingly ParA also associates with Wag31/DivIVA, a protein that dictates polar growth in Streptomyces and *Mycobacterium smegmatis* (Hempel et al., 2008; Ginda et al., 2013).

## Walker a Type Cytoskeletal ATPases in Archaeal DNA Segregation

Recent work on Archaea, the third branch of life, has led to a better understanding of the cellular functions and intracellular dynamics of cytoskeletal proteins in this group. In the thermophilic crenarchaeon *Sulfolobus solfataricus, segA* encodes for a ParA-like protein with a weak ATPase activity (She et al., 2001; Schumacher et al., 2015). SegB, on the other hand, is an archaeon specific protein that shares homology with proteins found in crenarchaea and euryarchaeal but has no sequence similarity to ParB or any other bacterial or eukaryotic proteins (Kalliomaa-Sanford et al., 2012). SegB is a DNA binding protein that binds specifically to palindromic sequences that constitute the centromeric sites, S1 and S2, found upstream of *segAB* genes in the *S. solfataricus* chromosome (Kalliomaa-Sanford et al., 2012; Barillà, 2016). While SegA assembles into polymers in the presence of ATP *in vitro*, SegB interacts with SegA in the presence of nucleotides and influences SegA polymerisation, which drives chromosome segregation (Kalliomaa-Sanford et al., 2012; Yen et al., 2021). Recent crystal structures of the SegA and SegB proteins complexed with DNA have revealed new insights into this archaeal DNA partitioning system.

Interestingly, SegA forms a non-canonical dimer that does not resemble the typical ATP-sandwich dimers observed in many ParA structures (Yen et al., 2021). Although SegB lacks any sequence similarity to bacterial or eukaryotic proteins (Schumacher et al., 2015), the structure of SegB reveals the presence of a ribbon-helix-helix (RHH) motif that is important for binding to the centromere-like DNA sites (Yen et al., 2021) and is reminiscent of many bacterial plasmid-encoded CBPs (Schumacher, 2008). Furthermore, SegB forms a superhelical chromatin-like structure and wraps around the DNA, giving rise to a left-handed helix as has been observed for the omega protein (ParB) of the *S. pyogenes* plasmid pSM19035. However, significant differences have been noted in regions that form the protein dimer interfaces (Weihofen et al., 2006; Soberón et al., 2011; Yen et al., 2021). Thus, the organization of archaeal chromosomal segregation genes and functions bear a close resemblance to bacterial *parABS* systems. Moreover, electron microscopy of SegA-SegB-DNA complexes in the presence of ATP revealed short oligomers that were rod-like or arc-shaped. The rod-like structures were only 30–40 nm in length. However, the formation of such oligomers only in the presence of ATP but not ADP suggests a role for SegA oligomers in segresome formation, chromosome organization and segregation in archaea (Yen et al., 2021).

Like many bacteria, archaea too rely on ParA proteins to segregate and maintain plasmids. pNOB8 is an archaeal plasmid from *Sulfolobus* and contains a unique partitioning system comprising three proteins and a centromeric site (She et al., 1998; Schumacher et al., 2015). While ParA_NOB8_ forms the NTPase motor protein with a Walker A motif, AspA is the centromere binding protein and thus performs the analogous function of a typical ParB. The archaeal plasmid, pNOB8, also carries an atypical ParB whose N-terminal domain shares homology with bacterial ParBs, but the C-terminal domain bears structural similarity to CENP-A, a eukaryotic protein involved in chromosome segregation (Schumacher et al., 2015). Nonetheless, ParB_NOB8_ acts as the adaptor protein that links the DNA to ParA_NOB8_ by binding to the AspA-centromere complex. AspA binds to the centromere sequence and creates a superhelical structure for ParB_NOB8_ binding. ParA_NOB8_ binds to this ParB-AspA-centromeric complex and facilitates the partitioning of the plasmid into the daughter cells (Schumacher et al., 2015). Recent crystal structures of ParA-AMPPNP-DNA complexes have revealed the presence of a multifaceted nsDNA binding site in ParA_NOB8_ (Zhang and Schumacher, 2017). The structural resemblance of the C-terminal domain of ParB_NOB8_ to CENP-A and its binding to ParA reveal a unifying theme that underlies the DNA segregation process in the three domains of life.

## Specialized Polar Tethering Proteins—Accessory Factors for ParA Mediated Chromosome Segregation in Bacteria

### Bactofilin

Bactofilins belong to a group of polymeric cytoskeletal proteins defined by a conserved DUF583 domain, also known as the bactofilin domain (Kühn et al., 2010; Punta et al., 2012). They are characterized by extended beta-sheet structures and are ubiquitous in both Gram-positive and Gram-negative bacteria (Lin and Thanbichler, 2013; Zuckerman et al., 2015). In the rod-shaped bacterium *Myxococcus xanthus*, four bactofilin paralogs have been identified. They serve diverse functions like cell-shape maintenance (Koch et al., 2011), cell motility *via* polar localization of a small GTPase, SofG (Bulyha et al., 2013) and chromosome segregation (Lin et al., 2017; Anand et al., 2020). Three paralogs of bactofilin, BacNOP assemble into a polymeric structure and form a complex with the ParB-like protein, PadC, to restrict ParABS machinery to a well-defined position in the subpolar region of the cell. The ParA_Mxa_ ATPase decorates the entire length of the bactofilin filament using the ParB-like adaptor protein PadC. The pole-distal ends of the bactofilin filaments are bound by the centromeric DNA (*parS*) binding protein ParB_Mxa_ ensuring that the genome is equitably transferred after each round of cell division (Lin et al., 2017; Anand et al., 2020).

### DivIVA

Discovered in *B. subtilis*, DivIVA was named so due to the defects in septum placement in its absence (Edwards et al., 2000; Perry and Edwards, 2006; Oliva et al., 2010; van Baarle et al., 2013). It is a tetrameric coiled-coil protein that binds to the membrane by sensing negative curvature (Lenarcic et al., 2009; Ramamurthi and Losick, 2009; Oliva et al., 2010) and is mainly found in Gram-positive bacteria (Lin and Thanbichler, 2013). During sporulation, DivIVA localizes to the poles of the cell and binds to RacA and Soj/SpoOJ (ParAB_Bsu_) complex (Wu and Errington, 2003; van Baarle et al., 2013). In addition, DivIVA associates with the peptidoglycan synthesis proteins in *Streptomyces, Corynebacterium* and *Mycobacterium*, organizes the growth directing tip-complex and interacts with ParB (Hempel et al., 2008, 2012; Donovan et al., 2012; Ginda et al., 2013; Holmes et al., 2013). Interestingly, a DivIVA bound protein, Scy, associates with the ParA ATPase in *S. coelicolor* (Ditkowski et al., 2013).

### PopZ

While DivIVA is restricted to Gram-positive bacteria, PopZ (polar organizer protein Z), an evolutionarily unrelated small, acidic protein with an alpha-helical structure present in many Gram-negative species of α-proteobacteria, serves a similar function (Bowman et al., 2008; Ebersbach et al., 2008; Holmes et al., 2016). PopZ has a highly conserved N-terminal and a C-terminal domain, analogous to DivIVA, required for higher-order assembly (Oliva et al., 2010; Bowman et al., 2013). PopZ assembles into polymers that form a network with gel-like properties, which is impermeable to macromolecules (Bowman et al., 2010). However, this 3D network of PopZ at the cell pole captures the ParA released from the nucleoid by the migrating ParB-*parS* complex. This capture of released ParA is mediated by direct interactions with PopZ (Ptacin et al., 2014). The increased concentration of ParA at the PopZ proximal new pole triggers the ParA DNA binding activity, which reinforces the movement of the ParB-*parS* complex in the same direction, prevents reversals and immobilizes the origins at cell poles *via* interactions with ParB. In *C. crescentus*, cells lacking PopZ have chromosome segregation defects, highlighting the important role played by PopZ in regulating ParA mediated chromosome segregation (Bowman et al., 2008, 2010; Ebersbach et al., 2008; Schofield et al., 2010; Ptacin et al., 2014; Holmes et al., 2016).

### TipN

In *C. crescentus*, TipN (Tip of New Pole) is a polarly localized landmark protein and contains a large C-terminal coiled-coiled domain and two transmembrane domains at its N-terminus. TipN relocalises at the cell division site in an FtsZ and FtsI dependent manner late during the pre-divisional stage and is thus present at the new pole in both the daughter cells (Huitema et al., 2006; Lam et al., 2006). At the new pole, TipN recruits TipF, a receptor for the second messenger c-di-GMP [bis-(3′-5′)-cyclic dimeric guanosine monophosphate] and a positive regulator of flagella assembly. However, this interaction between TipN and TipF is restricted in the swarmer cells due to the low level of c-di-GMP but allows the formation of a “flagella organizing center” at the new pole of the stalked cell due to the high levels of c-di-GMP in stalked cells (Davis et al., 2013). Interestingly, while Δ*tipN* cells exhibit mild chromosome segregation defects (Ptacin et al., 2010), deletion of *popZ* leads to synthetic lethality in Δ*tipN* cells due to severe DNA segregation and cell division defects (Schofield et al., 2010). TipN directly interacts with ParA_Ccr_ at the new pole and regulates the movement and overall speed of the ParB-*parS* complex (Ptacin et al., 2010; Schofield et al., 2010).

## Diverse Biological Functions of ParA Family of ATPases

**Figure 5:**
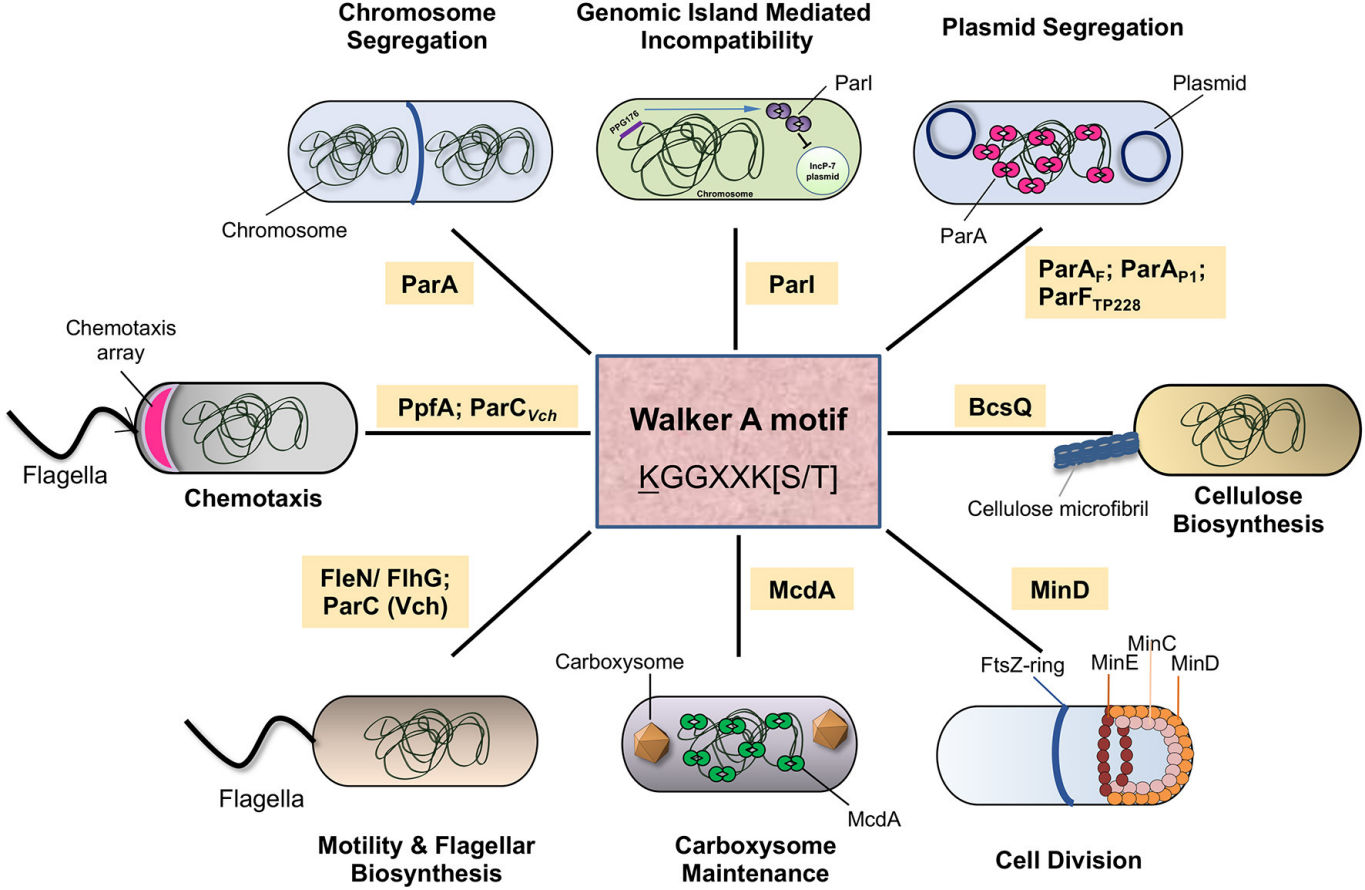
The diverse functions of the ParA family of ATPases in bacteria. The schematic representation depicts the various cellular functions performed by the ParA superfamily of ATPases in bacteria. Chromosome segregation is carried out by ParA carried in bacterial chromosomes. Plasmid segregation is carried out by ParA_F_ (in F plasmid) or ParA_P1_ (P1 bacteriophage) ParF_TP228_ (TP228, a plasmid from *S. newport*), FleN/FlgH regulates flagellar biosynthesis. McdA plays a role in carboxysome maintenance, and MinD controls cell division site positioning. ParI, an orphan ParA, regulates genomic island mediated incompatibility. BcsQ helps in cellulose biosynthesis. PpfA and ParC_Vch_ (ParA-like ATPase in *V. cholerae*) control polar localization of chemotaxis clusters in *Rhodobacter* and *Vibrio*, respectively.

Proteins with the deviant Walker A motif, or the P-loop, serve diverse functions in different life forms (Walker et al., 1982; Koonin, 1993). These include DNA replication and partitioning, cell cycle and division, subcellular localization and spatial organization. Examples include the ParA family of proteins found in bacterial genomes and plasmids, which play a role in DNA segregation. A few that are involved in cell division, like MinD and MipZ have been extensively reviewed (Motallebi-Veshareh et al., 1990; Ebersbach and Gerdes, 2005; Leonard et al., 2005b; Hayes and Barillà, 2006; Michie and Löwe, 2006; Thanbichler and Shapiro, 2006; Schumacher, 2007; Du and Lutkenhaus, 2012; Lutkenhaus, 2012; Barillà, 2016; Jalal and Le, 2020). At the same time, some others play an important role in positioning large macromolecular complexes such as carboxysomes within cells (Savage et al., 2010). These deviant Walker A motif-containing proteins are found in all life forms, ranging from Archaea to Bacteria and constitute a versatile system to build the spatial organization in biological systems (Figure 5). Thus, members of the ParA superfamily are not only involved in plasmid and chromosome partitioning but are also involved in the maintenance of other cellular cargo in many bacteria. We outline a few examples here that highlight the diversity of this superfamily of proteins within the domain Bacteria.

### MinD—A Spatial Regulator of Cell Division Site

MinD, a component of the *min* system, is a Walker A ATPase member majorly recruited for regulating cell division in bacteria (Lutkenhaus and Sundaramoorthy, 2003; Lutkenhaus, 2012). The structure of the MinD dimer reveals its similarity to the ParA proteins such as Soj (ParA_Bsu_) (Wu et al., 2011). The *min* locus consists of two other genes, *minC* and *minE*, wherein MinC functions as an inhibitor of FtsZ ring assembly *via* its interaction with MinD (Hu et al., 1999; Hu and Lutkenhaus, 2000; Dajkovic et al., 2008). MinD, along with MinE, undergoes pole-to-pole oscillations and produces a gradient of MinCD division inhibitory complex (Hu and Lutkenhaus, 1999; Raskin and de Boer, 1999; Hale et al., 2001; Meinhardt and de Boer, 2001) with the maximum time-averaged concentration at the cell poles (Lutkenhaus, 2012). MinD, in its ATP bound dimeric form, binds to the membrane *via* a C-terminal amphipathic helix (Hu et al., 2002; Szeto et al., 2002; Hu and Lutkenhaus, 2003; Zhou and Lutkenhaus, 2003). Following this membrane association, MinE is recruited, which stimulates the ATPase activity of MinD and thus releases it from the bacterial membrane (Hu and Lutkenhaus, 2001; Hu et al., 2002; Park et al., 2011; Wu et al., 2011). Crystal structures of the MinD-MinE complexes reveal the conformational changes in MinE upon interaction with MinD. The structures also show how two released β-sheets in a four-stranded β-sheet MinE dimer convert into α-helices and suggests how MinE remains associated with the MinD-membrane complex (Park et al., 2011). Repeated binding and release cycles of MinD driving oscillation of MinCD complex that acts as a spatial regulator of FtsZ ring in bacteria. The period of the oscillations is dictated by the built-in delays in the system, such as nucleotide hydrolysis and exchange rates in MinD (Howard et al., 2001; Meinhardt and de Boer, 2001; Huang et al., 2003; Kretschmer and Schwille, 2016). Numerous studies have reconstituted the dynamics of the MinDE system *in vitro* on supported membrane bilayers and observed standing waves of MinD being chased by MinE and recapitulate oscillations within confinement (Loose et al., 2008, 2011; Ivanov and Mizuuchi, 2010; Zieske and Schwille, 2013, 2014; Vecchiarelli et al., 2016).

Further, the deletion of MinD results in the production of anucleate cells suggesting that MinD plays a critical role in chromosome segregation (Di Ventura et al., 2013). Numerical computer simulations have proposed that the *min* system may contribute to the movement of the chromosome from the mid-cell to the poles and might be mediated by the binding of MinD to DNA (Di Ventura et al., 2013). However, recent *in vitro* reconstitution experiments of MinDE on membrane bilayers failed to detect any direct recruitment of DNA to MinD, suggesting that the chromosome segregation defects in the absence of Min proteins might be due to indirect effects (Ramm et al., 2018).

### McdA—ParA-Like Proteins in Carboxysome Maintenance in Cyanobacteria

Carboxysomes are membrane-bound organelles in Cyanobacteria that help in carbon fixation. These proteinaceous micro-compartments exist in low-copy numbers in the cells and thus depend on partitioning machinery to ensure their transmission during cell division (MacCready et al., 2018, 2020). Fluorescent labeling of the nucleoid, carboxysomes and McdA inside these cells has enabled the tracking of carboxysomes in live cells. Fluorescence imaging of carboxysomes has shown that these organelles are arranged linearly and segregate equally during cell division that was dependent upon a ParA homolog in Synechococcus elongatus (Savage et al., 2010). Recent studies have further shown that the ParA-like Walker A ATPase partitioning machinery, now named McdA (for Maintenance of Carboxysome Distribution), mediates Carboxysome maintenance (MacCready et al., 2018). Although McdA lacks the signature amino-terminal lysine residue (Table 1), it has a strong ATPase and non-specific DNA binding activity. Moreover, another small protein McdB, unrelated to ParB, has been shown to stimulate the ATPase activity of McdA and regulate carboxysome positioning. Although McdB shows no sequence similarity to ParB, it can form higher-order oligomers like ParB (Schumacher et al., 2019). McdB (like ParB) stimulates ATPase activity of McdA driving the directed movement of carboxysome toward a higher concentration of McdA on the nucleoid by a diffusion ratchet mechanism. Thus, Carboxysomes also employ a McdAB protein complex in a manner very similar to the ParAB complex (MacCready et al., 2018).

### PpfA—An Orphan ParA Promoting Chemoreceptor Cluster Formation

Certain *par* loci in bacterial chromosomes contain only *parA* sequences and lack *parB* and centromeric sequence *parS*. These additional *par* loci are located outside the *parAB* operon (found close to the *oriC*) and are referred to as orphan ParA systems. These orphan ParAs can be found in many metabolic operons of bacterial genomes. Also, reports suggest that bacterial genomes encode multiple orphan ParAs. One such orphan ParA, called PpfA, is involved in chemotactic signaling in *Rhodobacter sphaeroides*. The chemotaxis protein cluster is formed by the partner proteins TlpT and CheW proteins. Mutants in the conserved Walker A motif are known to affect cluster formation. Thus, PpfA helps in the dispersion and segregation of the chemoreceptor clusters (Thompson et al., 2006; Roberts et al., 2012). Remarkably, a distinct clade of ParA-like ATPase is encoded within the chemotaxis operon in *V. cholerae* (named ParC) and many γ-proteobacteria that have polar flagella (Ringgaard et al., 2011). In *V. cholerae*, the ParA-like ATPase, ParC, regulates the polar localization of the chemotaxis proteins CheW1 and CheY3 (Ringgaard et al., 2011). The subcellular localization dynamics of ParC are altered by its binding partner, ParP, which promotes the polar assembly of the chemotaxis array (Ringgaard et al., 2011, 2018; Alvarado et al., 2017).

### ParI—An Incompatibility Factor Residing on a Genomic Island

ParI is an Orphan ParA member of the Walker A ATPase family present in the genomic island of *Pseudomonas putida* (Miyakoshi et al., 2012). The expression of ParI negatively regulates the maintenance of IncP-7 plasmids and results in their loss from cells. Studies on ParI have revealed that mutations in the conserved Walker A motif region (mainly the ATPase domain) of ParI fail to destabilize IncP-7 plasmids. ParI is an example of plasmid-mediated incompatibility residing within a genomic island.

### BscQ—An Orphan ParA Involved in Cellulose Biosynthesis

Bacteria produce cellulose as a biofilm matrix polymer to enable the cohesion of biofilms. BcsQ (bacterial cellulose synthesis) proteins help produce cellulose in enterobacteria. BcsQ is a homolog of the ParA/MinD family of ATPase and is activated by cyclic di-GMP mediated signalling. Using fluorescently tagged BcsQ, it has been confirmed that this protein mainly localizes to the cell poles in bacteria and cell-cell adhesion mainly occurs *via* cellulose production at the cell poles. Thus, a ParA/MinD family of ATPase controls cell-cell adhesion and biofilm formation by regulating cellulose biosynthesis (Le Quéré and Ghigo, 2009).

### FleN/FlhG/MinD2—A ParA Protein Regulating Flagellar Biosynthesis

Flagella serves as a locomotory organ in many organisms. The bacterial flagella is a complex structure requiring ~40 genes for its assembly (Dasgupta et al., 2003). The flagellar genes in bacteria are involved in locomotion, biofilm formation, and pathogenesis (O'Toole and Kolter, 1998; Gellatly and Hancock, 2013; Guttenplan and Kearns, 2013; Mukherjee and Kearns, 2014). The flagella number and distribution are characteristic features of each organism. In *P. aeruginosa*, the number of flagella is regulated by FleN (Dasgupta et al., 2000, 2003; Köhler et al., 2000; Dasgupta and Ramphal, 2001; van Ditmarsch et al., 2013). FleN, also called FlhG or MinD2, is a ParA/MinD superfamily protein (Table 1), whose absence leads to the upregulation of genes involved in the synthesis of the flagellar motor, basal body, hook proteins etc. and conferring the multi-flagellate phenotype (Dasgupta et al., 2003; Chanchal et al., 2017). FleN acts antagonistic to FleQ and is known to inhibit FleQ dependent transcription. Such effect is essential for maintaining the correct number of flagella in the cell (Dasgupta and Ramphal, 2001; Chanchal et al., 2017). Homologs of FleN are also present in many other bacteria, including *B. subtilis, V. cholerae, Geobacillus thermodenitrificans*, and *Campylobacter jejuni* (Correa et al., 2005; Guttenplan and Kearns, 2013; Schuhmacher et al., 2015; Gulbronson et al., 2016).

## Concluding Remarks

Central to all life is the duplication and equal partitioning of the genetic material into the daughter cells. Studies over the last century have revealed that complex dynamic structures such as the spindle microtubules assembled during a specific time in the cell cycle govern these processes in eukaryotic cells. On the contrary, how simple unicellular bacterial cells achieve DNA partitioning has remained elusive for a long time. It is now appreciated how several bacterial genomes and multi-drug resistance carrying plasmids, including the historic Fertility factor or the F plasmid, utilize a Walker A class of cytoskeletal ATPases (WACA) protein ParA (present in Bacteria and Archaea) to partition DNA. While ParA functions as an ATP-dependent motor protein, ParB constitutes the adaptor protein that stimulates the ATPase activity ParA and drives the dynamicity of the system.

Studies on various members of the ParA superfamily of proteins have established that the binding of ParA to non-specific DNA is vital for its function in plasmid maintenance. A chemophoretic gradient of ParA across the bacterial chromosome (nsDNA) is thought to drive the unidirectional movement of the plasmid DNA toward the cell poles. The nsDNA binding activity of ParA itself is regulated by ATP binding and a conformational switch. In the future, it would be important to establish the existence of the conformational switch to the ParA-ATP^*^ state for multiple members of the ParA family of proteins. Further, deciphering the structural changes associated with the conformational switch and kinetics of substrate binding should lead to a better understanding of ParA binding to nsDNA and stimulation of its ATPase activity by ParB. Overall, the structures of ParA proteins are highly conserved, and a unified molecular understanding of the mechanism by which ParA protein functions has emerged. However, given the wide range of cellular functions and evolutionary divergence, it would be fascinating to probe the subtle differences in the molecular mechanisms employed by various ParA proteins.

Moreover, recent EM studies of the ParA2_Vch_ and archaeal SegAB complex revealing a DNA-ParA filament complex are compelling and have reignited the idea that ParA functions require its polymerisation or oligomerisation (Parker et al., 2021; Yen et al., 2021). These studies could revive the polymeric cytoskeleton models proposed for ParA mediated DNA partitioning, especially given the earlier observations on ATP-dependent aggregation or polymerisation of some ParA members (Ebersbach and Gerdes, 2001; Suefuji et al., 2002; Leonard et al., 2004, 2005a; Barillà et al., 2005, 2007; Lim et al., 2005; Bouet et al., 2007; Dunham et al., 2009; Schumacher et al., 2012; Volante and Alonso, 2015). While the physiological relevance of such polymeric structures formed by ParA *in vitro* is still debated, unequivocal evidence for the existence of such polymeric or oligomeric structures of ParA *in vivo* could be challenging. However, experiments using mutants that have distinct activities of nsDNA binding and polymerisation might help reveal the role of polymerisation/oligomerisation in ParA functions. Moreover, given the overarching conserved design in the structure of the ParA proteins, subtle changes to the structure and conformation might drive these diverse mechanisms. Extensive biochemical and structural studies leveraging the advances in single-molecule techniques, single-particle and cellular correlative-electron microscopy should reveal the underpinnings of mechanisms by which the ParA superfamily of proteins function across domains of life.

It is evident that ParA proteins are vital for bacterial survival and maintenance of virulence factors and antibiotic resistance on plasmids. Since the ParA family of proteins is unique to bacteria and archaea, they could potentially be considered targets for developing new antibiotics. Insights into subtle differences in the interactions of ParA proteins with nsDNA, ParB and itself could be significant for such studies. However, a major challenge could be the development of specific assays and high-throughput screening platforms. The positive selection assay for loss of plasmids developed by Swaine Chen's group could be a way forward (Chen et al., 2017). Understanding the evolution of variations and diverse mechanisms in ParA functions could also be leveraged in synthetic biology applications to design and dictate spatial organization in artificial cell-like systems.

Finally, the recent findings on the CTP binding, hydrolysis and formation of LLPS condensates by ParB have indeed added an entirely new dimension and complexity to our understanding of the plasmid and chromosomal segregation mechanisms. It will be interesting to see the influence of ParB-CTP on ParA functions, especially on the conformational changes to the proposed ParA-ATP^*^ state, nucleoid association and dynamics and polymerisation. Reconstitution experiments and *in vivo* live-cell imaging at high-resolution of the segresome complex from diverse species, together with structural studies employing cryo-electron microscopy and cellular tomography, should help provide more insights into the functioning of these amazing and unique family of proteins.

## Author Contributions

DM wrote the initial draft. RS wrote sections of the manuscript and revised the manuscript. DM and RS edited the manuscript. Both authors contributed to the article and approved the submitted version.

## Conflict of Interest

The authors declare that the research was conducted in the absence of any commercial or financial relationships that could be construed as a potential conflict of interest.

## Publisher's Note

All claims expressed in this article are solely those of the authors and do not necessarily represent those of their affiliated organizations, or those of the publisher, the editors and the reviewers. Any product that may be evaluated in this article, or claim that may be made by its manufacturer, is not guaranteed or endorsed by the publisher.
